# Supplementary material for: Time Management and Task Prioritization Curriculum for Pediatric and Internal Medicine Subinternship Students
Source: MedEdPORTAL. 2022 Feb 22;18:11221. doi: 10.15766/mep_2374-8265.11221 (PMC8861138; doi:10.15766/mep_2374-8265.11221)
Supplement: Supplementary file 1 — Student Survey Evaluations.docxPreworkshop Exercise for Pediatric Students.docxPreworkshop Exercise for Internal Medicine Students.docxWorkshop for Pediatric Students.pptxWorkshop for Internal Medicine Students.pptxSpeaker Notes for Workshop.docx [file mep_2374-8265.11221-s001.zip › C. Preworkshop Exercise for Internal Medicine Students.docx]

**Time Management Exercise for Internal Medicine Sub-Internship Students**

1. **What tasks do you need/want to accomplish each day as an Internal Medicine Sub-Intern taking care of 2-3 patients?** Think about clinical responsibilities as well as personal responsibilities. For example - finishing notes, calling consults, putting in orders, calling a nurse back, going to rest room, eating a meal/snack, etc.)


1. **Rate the importance of each of the tasks in Question 1 in terms of prioritization for your day. Which one of these needs to happen first vs which ones can wait until later? Use the Time Management Matrix Technique (TMMT) below.**

|  | **Important** | **Less Important** |
| --- | --- | --- |
| **Urgent** |  |  |
| **Less Urgent** |  |  |

**Now let’s translate your TMMT priorities to patient cases. You arrive to work and receive sign out on several patients. The information you receive on each patient is detailed below. Please read the cases and answer the following questions.**

**Patient 1:** John Hurts is a 61 year old man with a past medical history of hypertension, GERD, and prior L4-5 hemilaminectomies and foraminotomies in 2017, who presented to the ED last night with localized injection site pain following caudal epidural steroid injection. Now with a concern for L5/S1 osteomyelitis. Will need biopsy today.

**Patient 2:** Jeanine Cook is a 54 year old woman with a past medical history of coronary artery disease (CAD)**,** non-small cell lung cancer (NSCLC) treated in 2012 who presented 2 days ago with acute kidney injury (AKI) with a Cr of 2.7 from baseline of 1.1. Furosemide was held and IV fluids were given. Repeat creatinine 2.3.

**Patient 3:** Dane Bryant is a 36 year old African American male with history of Hemoglobin SS Disease on monthly exchange transfusions with history of proteinuria and retinopathy who is hospitalized with vaso-occlusive crisis 4 days ago. Hemoglobin has been stable. Chest imaging is clear with no current oxygen needs. He has been stable on IV pain medications via PCA or patient-controlled analgesia for last 24 hours.

**Patient 4:** Elizabeth Clark is a 27 year old female who has a history of IVDU who was admitted with MRSA bacteremia. She has been stable for last 5 days on Vancomycin and has a PICC line. She is normally seen by the intern who is off today. Social work paged that transport will get her at 10:30 AM to a skilled nursing facility. None of the discharge work (medication reconciliation, discharge summary) has been done.

**Patient 5:** Gerald Mann is a 72 year old male with a past medical history of remote pituitary dysgerminoma status post resection and radiation treatment, panhypopituitarism, anaplastic astrocytoma in the left frontal cortex status post resection and chemoradiation therapy in 2017, DVT/PE on Xarelto, and cerebrovascular accident (CVA) in 2012 complicated by residual balance difficulty and dysarthria who presented two days ago with altered mental status (AMS). There does not seem to be an organic cause of this currently so you are waiting for him to return to baseline.

**Patient 6:** Ariel Slater is a 48 year old woman past medical history of hypertension and type 2 diabetes who presented with chest pain. Patient has been ruled out for myocardial infarction (MI) and is currently without chest pain. Patient is on all her home medications and has no symptoms. The plan is to discharge her home today.

**You have received sign-out on your patients in the morning and are planning your time before attending rounds. What are some ways you can be time efficient during *pre-rounding*?**


**You have finished rounding on your patients in the morning and trying to think what you could do before attending round start in the next 30 minutes. Tasks can include finishing notes, calling consults, putting in orders, evaluating a particular patient, calling a nurse back, going to rest room, eating a meal/snack, etc. Use the TMMT matrix.**

|  | **Important** | **Less Important** |
| --- | --- | --- |
| **Urgent** |  |  |
| **Less Urgent** |  |  |

**Rounds are done at 11 AM, now what would you prioritize?**
