# Supplementary material for: Time Management and Task Prioritization Curriculum for Pediatric and Internal Medicine Subinternship Students
Source: MedEdPORTAL. 2022 Feb 22;18:11221. doi: 10.15766/mep_2374-8265.11221 (PMC8861138; doi:10.15766/mep_2374-8265.11221)
Supplement: Supplementary file 1 — Student Survey Evaluations.docxPreworkshop Exercise for Pediatric Students.docxPreworkshop Exercise for Internal Medicine Students.docxWorkshop for Pediatric Students.pptxWorkshop for Internal Medicine Students.pptxSpeaker Notes for Workshop.docx [file mep_2374-8265.11221-s001.zip › D. Workshop for Pediatric Students.pptx]

## Slide 1
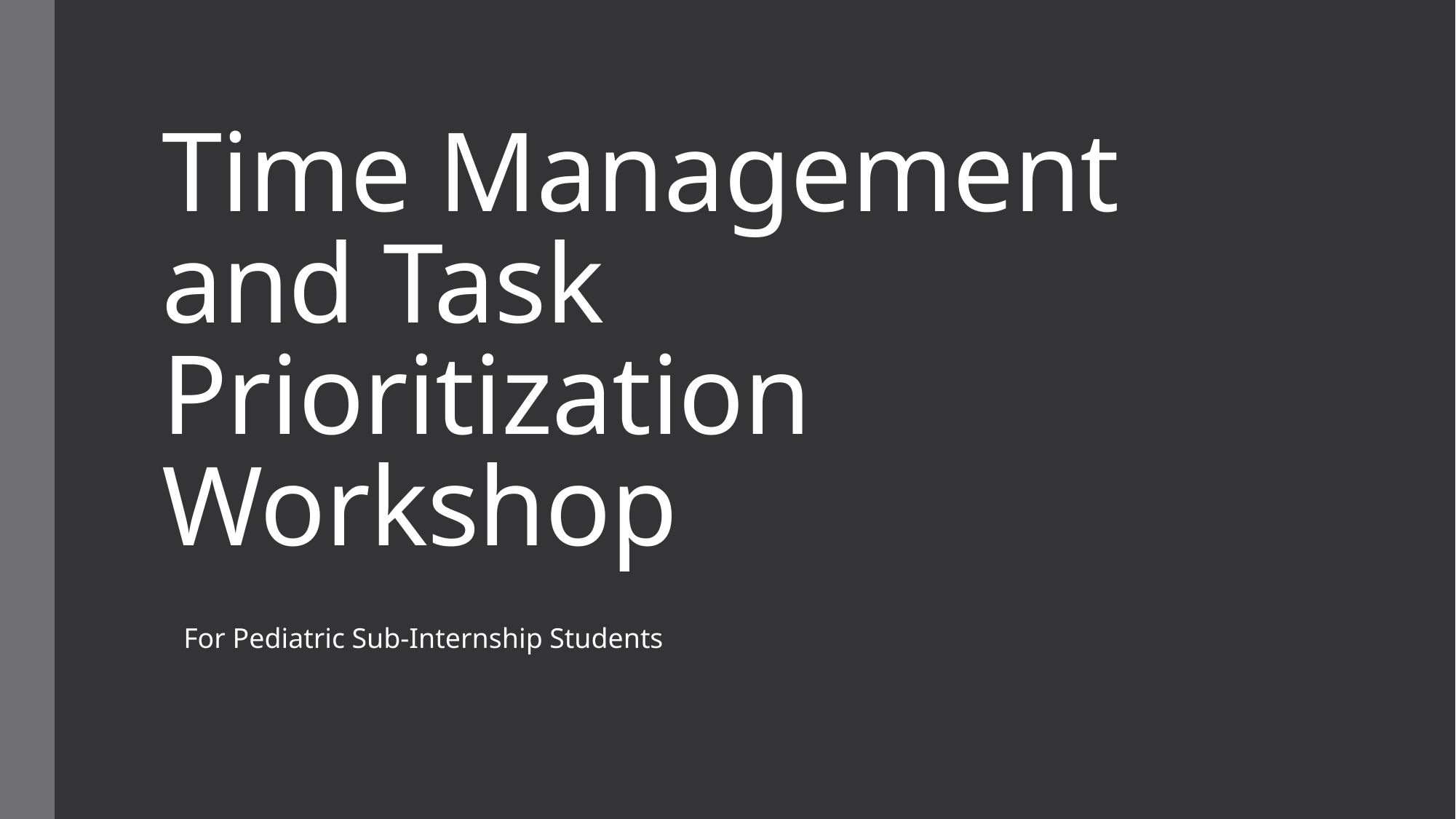

# Time Management and Task Prioritization Workshop
For Pediatric Sub-Internship Students

## Slide 2
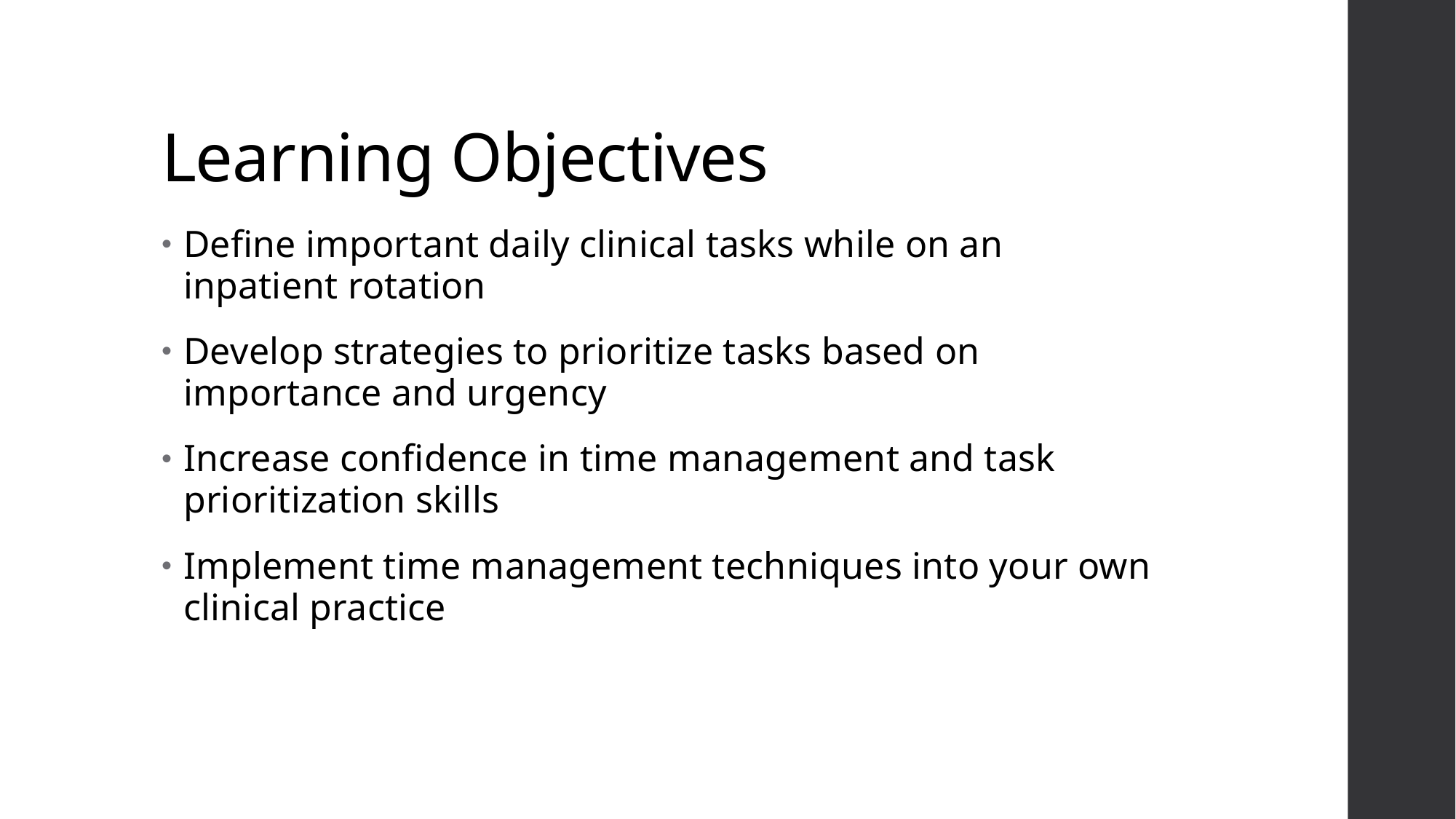

# Learning Objectives
Define important daily clinical tasks while on an inpatient rotation
Develop strategies to prioritize tasks based on importance and urgency
Increase confidence in time management and task prioritization skills
Implement time management techniques into your own clinical practice

## Slide 3
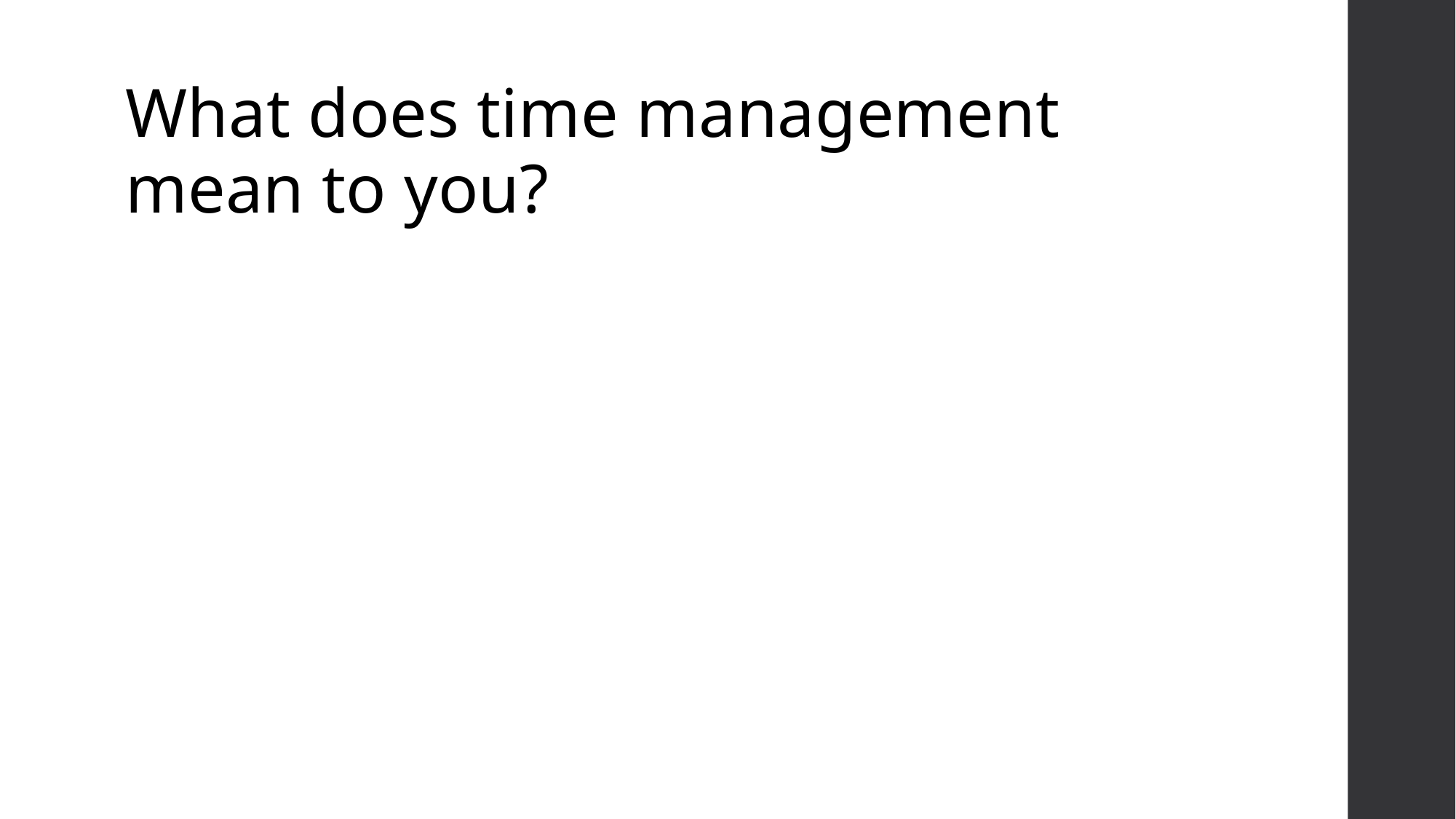

What does time management mean to you?

## Slide 4
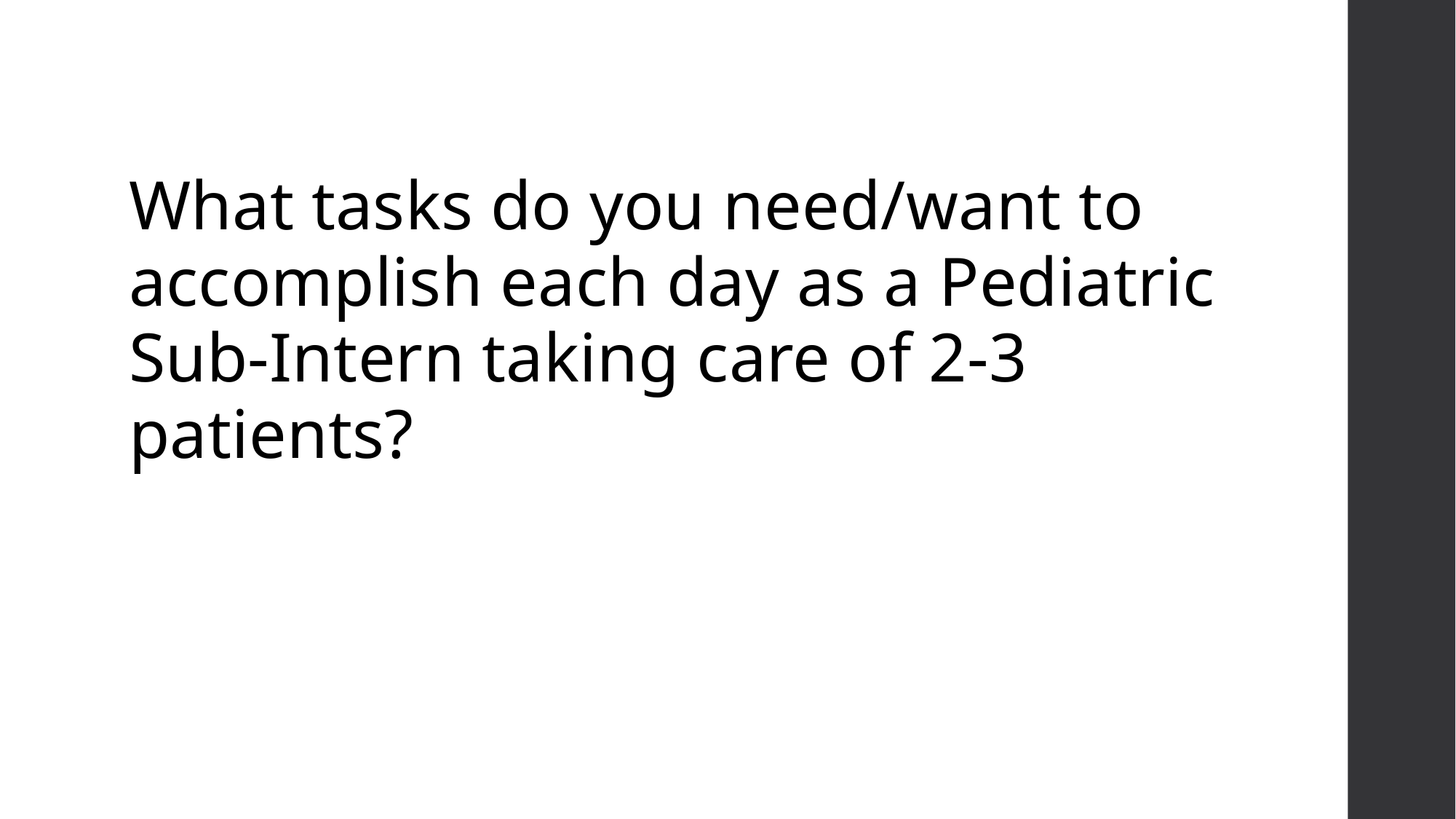

What tasks do you need/want to accomplish each day as a Pediatric Sub-Intern taking care of 2-3 patients?

## Slide 5
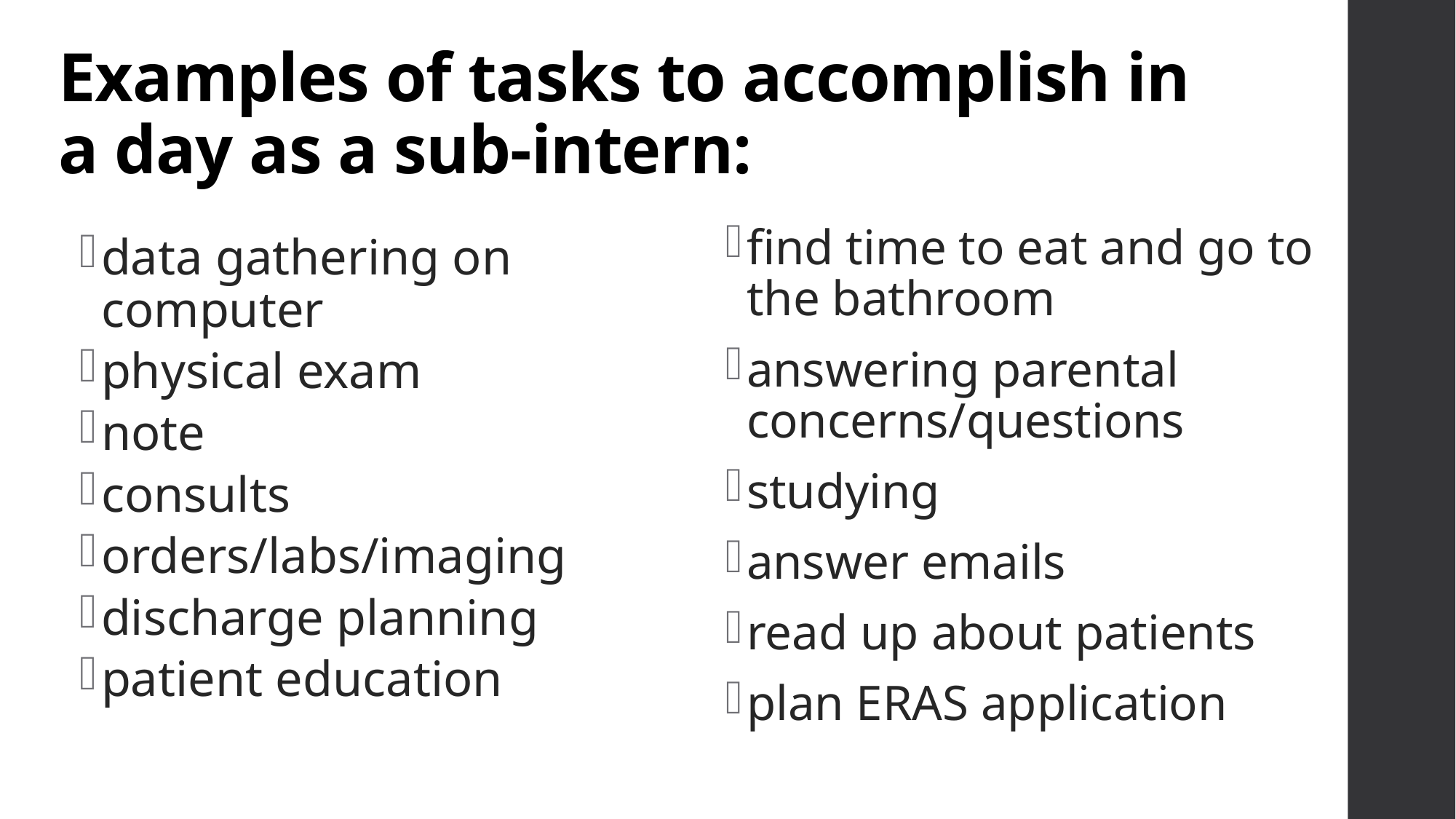

# Examples of tasks to accomplish in a day as a sub-intern:
find time to eat and go to the bathroom
answering parental concerns/questions
studying
answer emails
read up about patients
plan ERAS application
data gathering on computer
physical exam
note
consults
orders/labs/imaging
discharge planning
patient education

## Slide 6
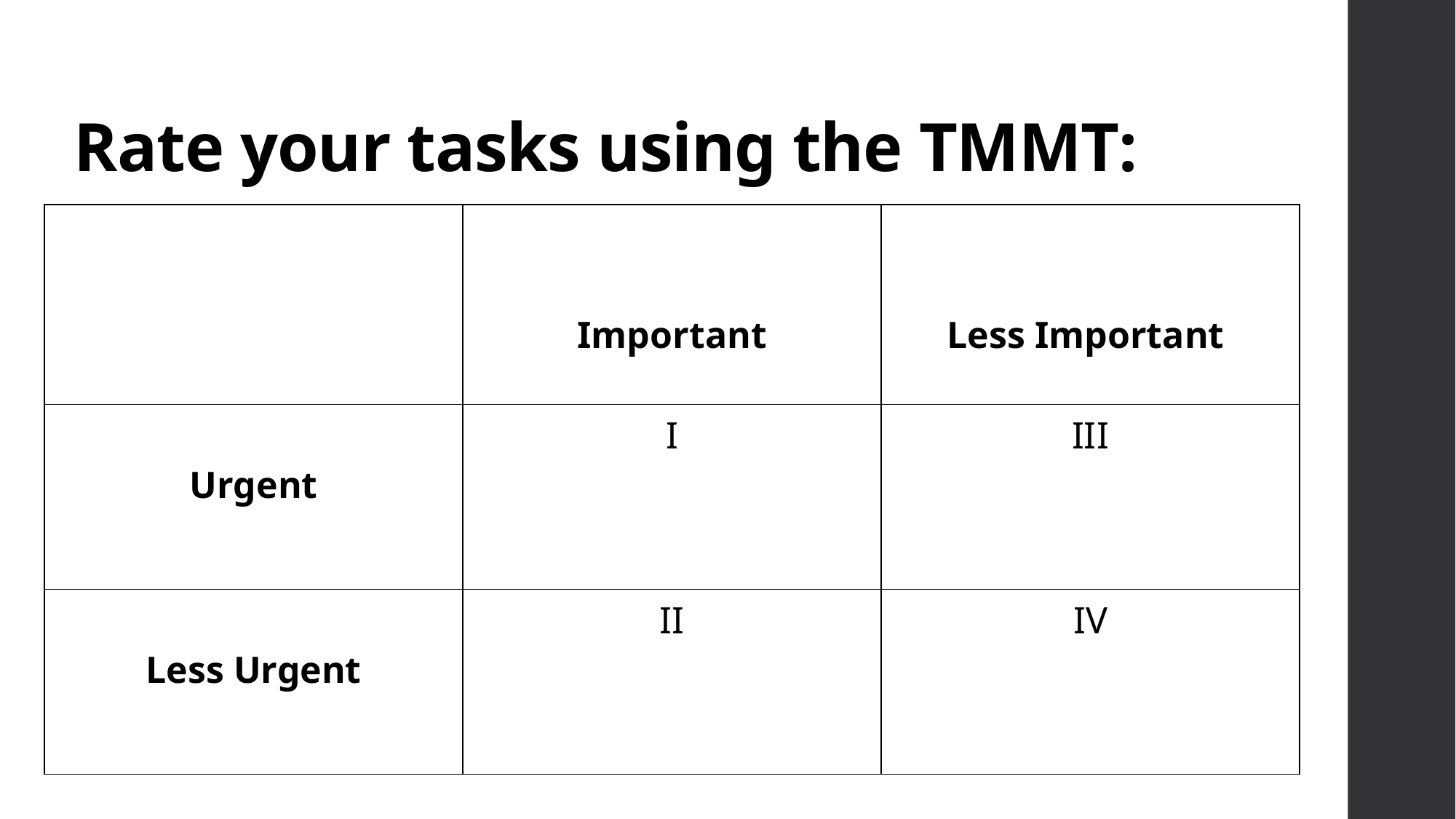

# Rate your tasks using the TMMT:
| | Important | Less Important |
| --- | --- | --- |
| Urgent | I | III |
| Less Urgent | II | IV |

## Slide 7
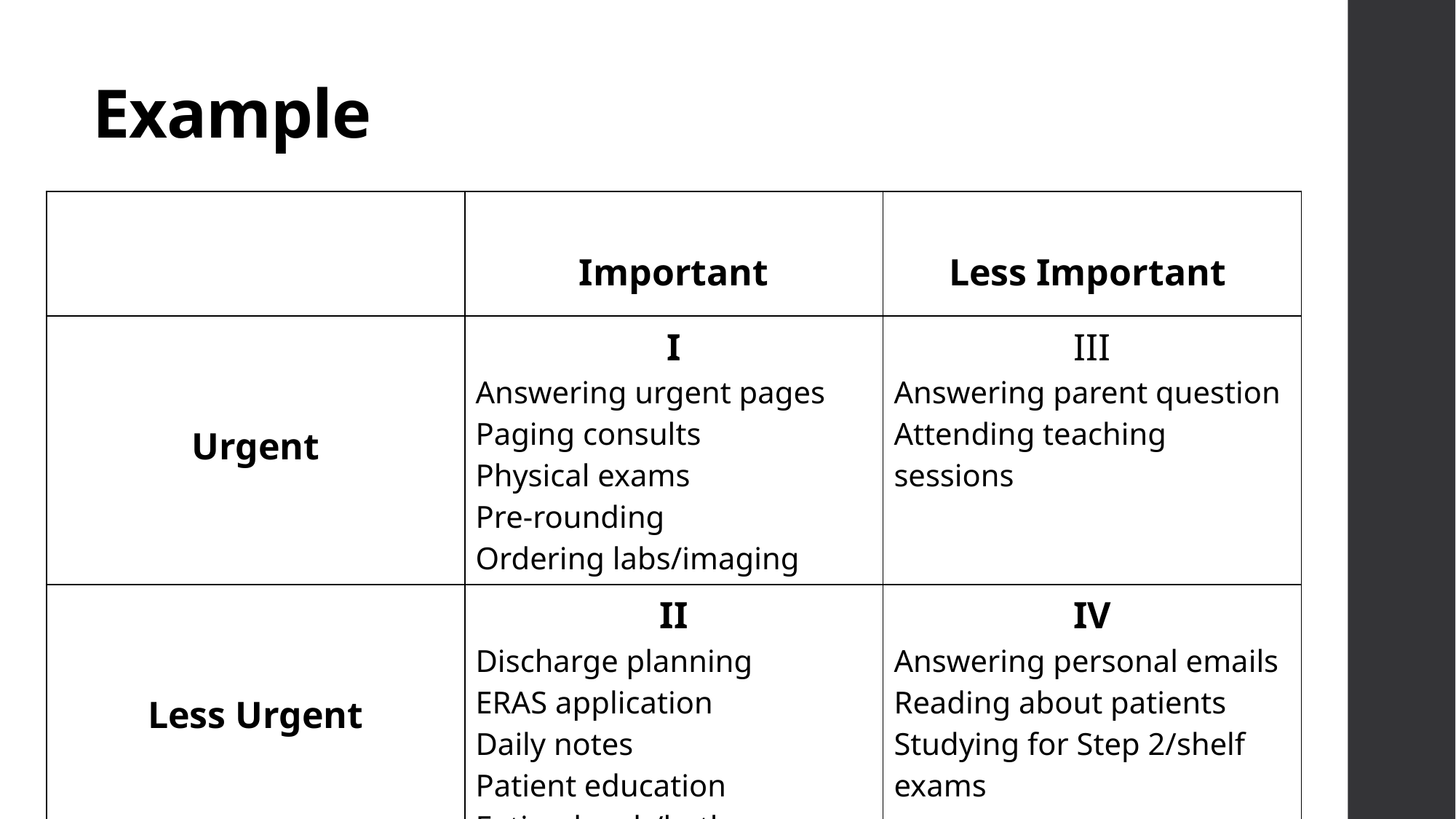

# Example
| | Important | Less Important |
| --- | --- | --- |
| Urgent | I Answering urgent pages  Paging consults Physical exams Pre-rounding Ordering labs/imaging | III Answering parent question Attending teaching sessions |
| Less Urgent | II Discharge planning ERAS application Daily notes Patient education Eating lunch/bathroom | IV Answering personal emails Reading about patients Studying for Step 2/shelf exams |

## Slide 8
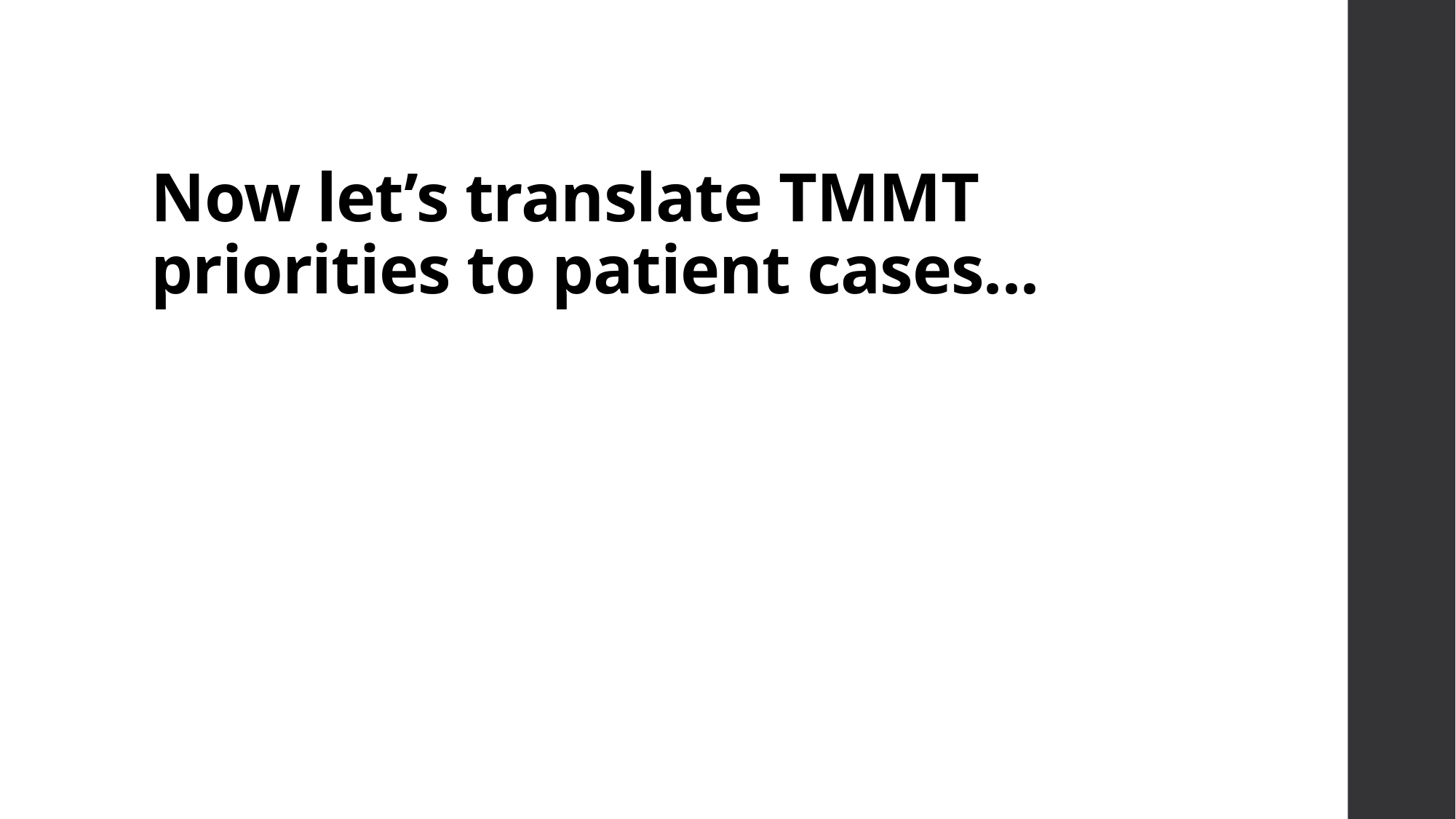

# Now let’s translate TMMT priorities to patient cases...

## Slide 9
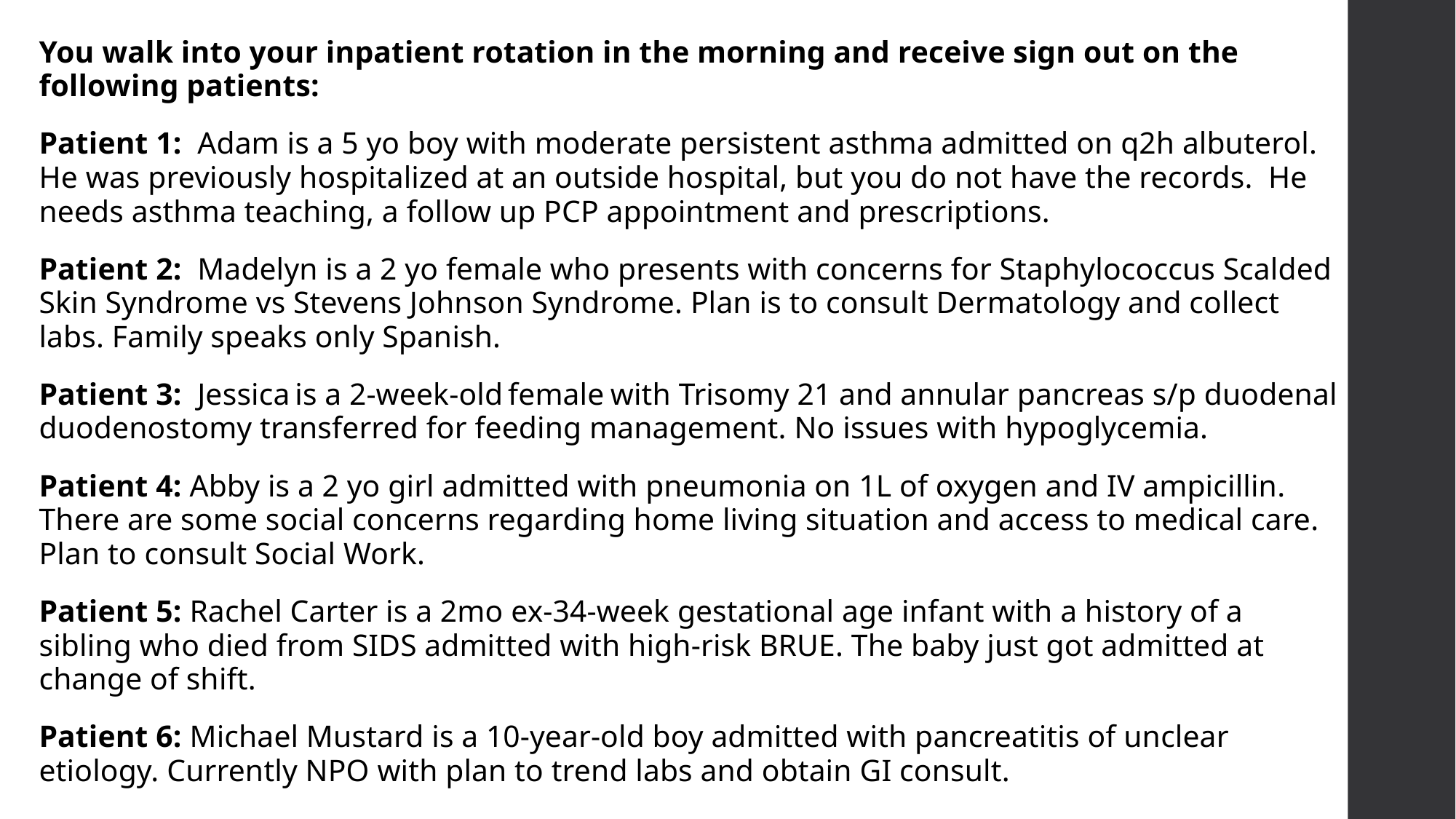

You walk into your inpatient rotation in the morning and receive sign out on the following patients:
Patient 1:  Adam is a 5 yo boy with moderate persistent asthma admitted on q2h albuterol.  He was previously hospitalized at an outside hospital, but you do not have the records.  He needs asthma teaching, a follow up PCP appointment and prescriptions.
Patient 2:  Madelyn is a 2 yo female who presents with concerns for Staphylococcus Scalded Skin Syndrome vs Stevens Johnson Syndrome. Plan is to consult Dermatology and collect labs. Family speaks only Spanish.
Patient 3:  Jessica is a 2-week-old female with Trisomy 21 and annular pancreas s/p duodenal duodenostomy transferred for feeding management. No issues with hypoglycemia.
Patient 4: Abby is a 2 yo girl admitted with pneumonia on 1L of oxygen and IV ampicillin. There are some social concerns regarding home living situation and access to medical care. Plan to consult Social Work.
Patient 5: Rachel Carter is a 2mo ex-34-week gestational age infant with a history of a sibling who died from SIDS admitted with high-risk BRUE. The baby just got admitted at change of shift.
Patient 6: Michael Mustard is a 10-year-old boy admitted with pancreatitis of unclear etiology. Currently NPO with plan to trend labs and obtain GI consult.

## Slide 10
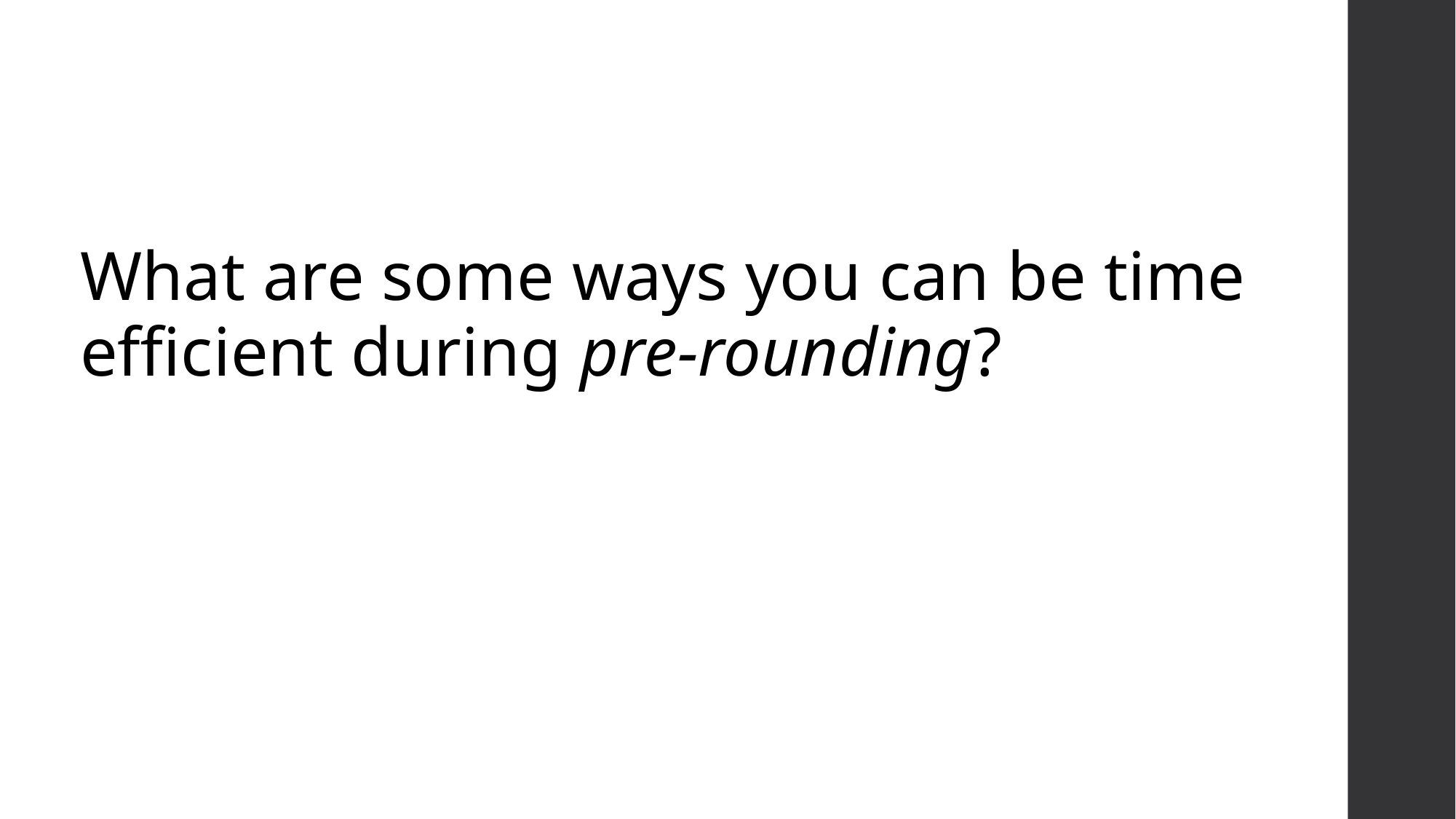

What are some ways you can be time efficient during pre-rounding?

## Slide 11
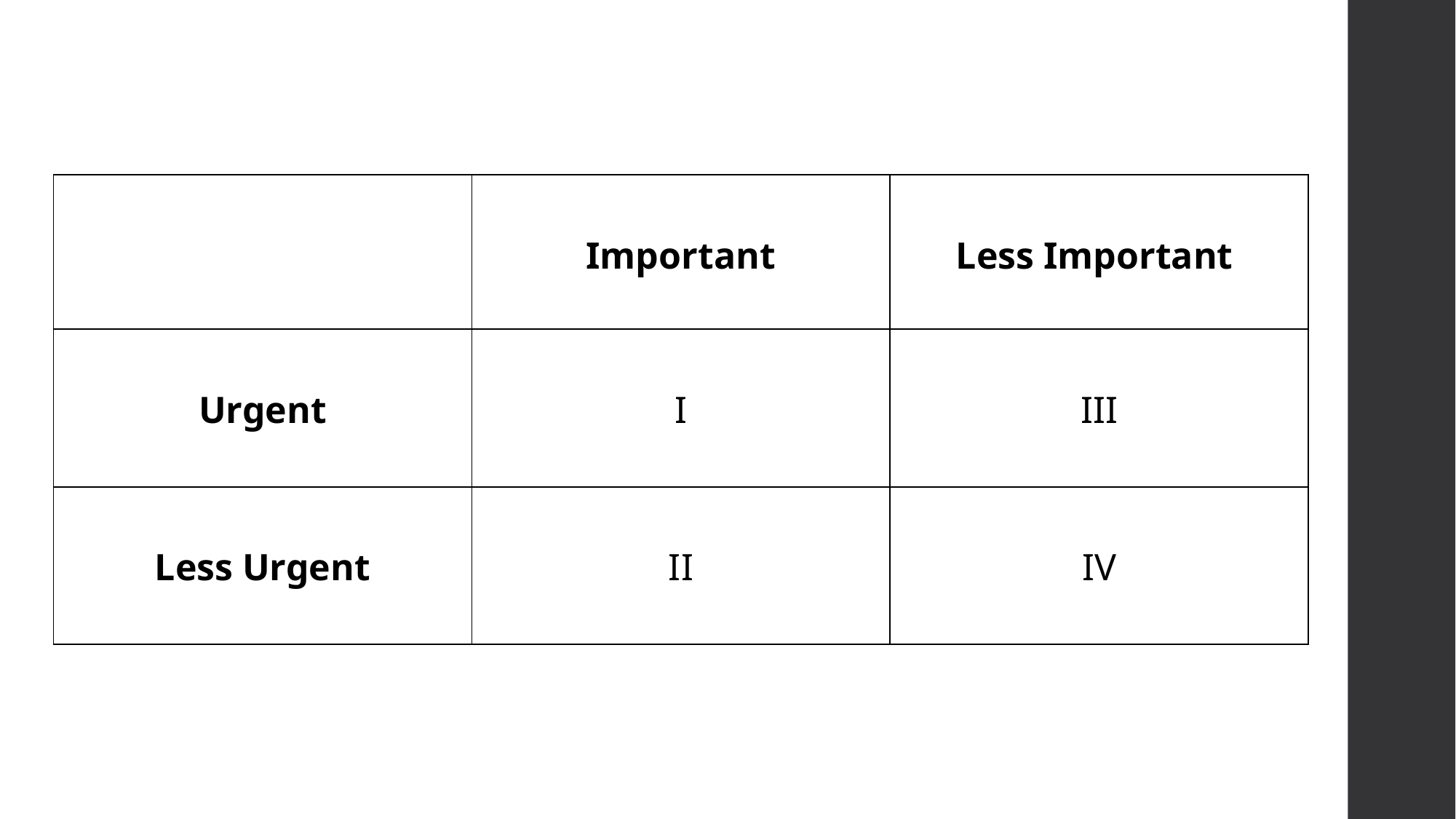

| | Important | Less Important |
| --- | --- | --- |
| Urgent | I | III |
| Less Urgent | II | IV |

## Slide 12
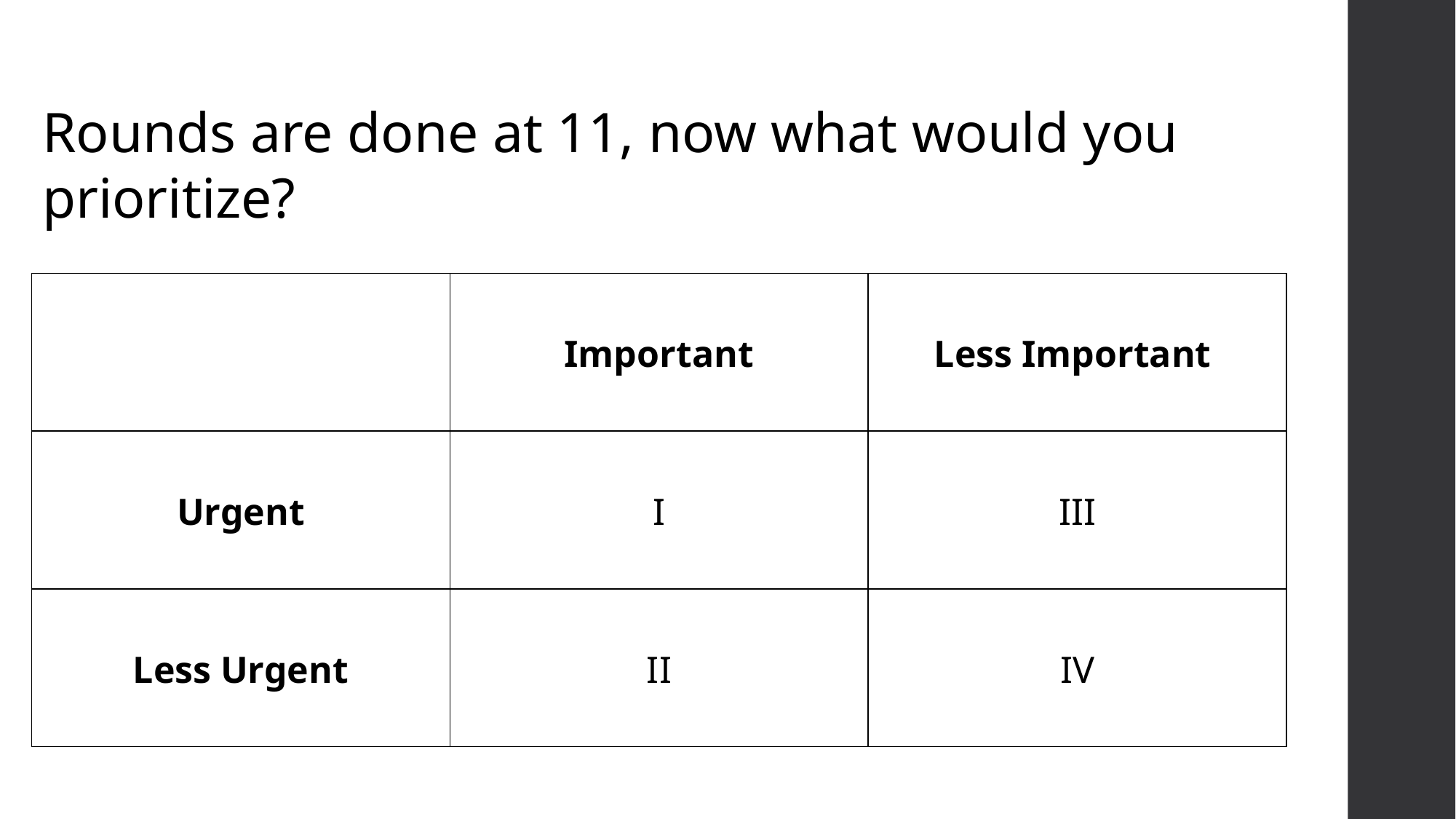

Rounds are done at 11, now what would you prioritize?
| | Important | Less Important |
| --- | --- | --- |
| Urgent | I | III |
| Less Urgent | II | IV |

## Slide 13
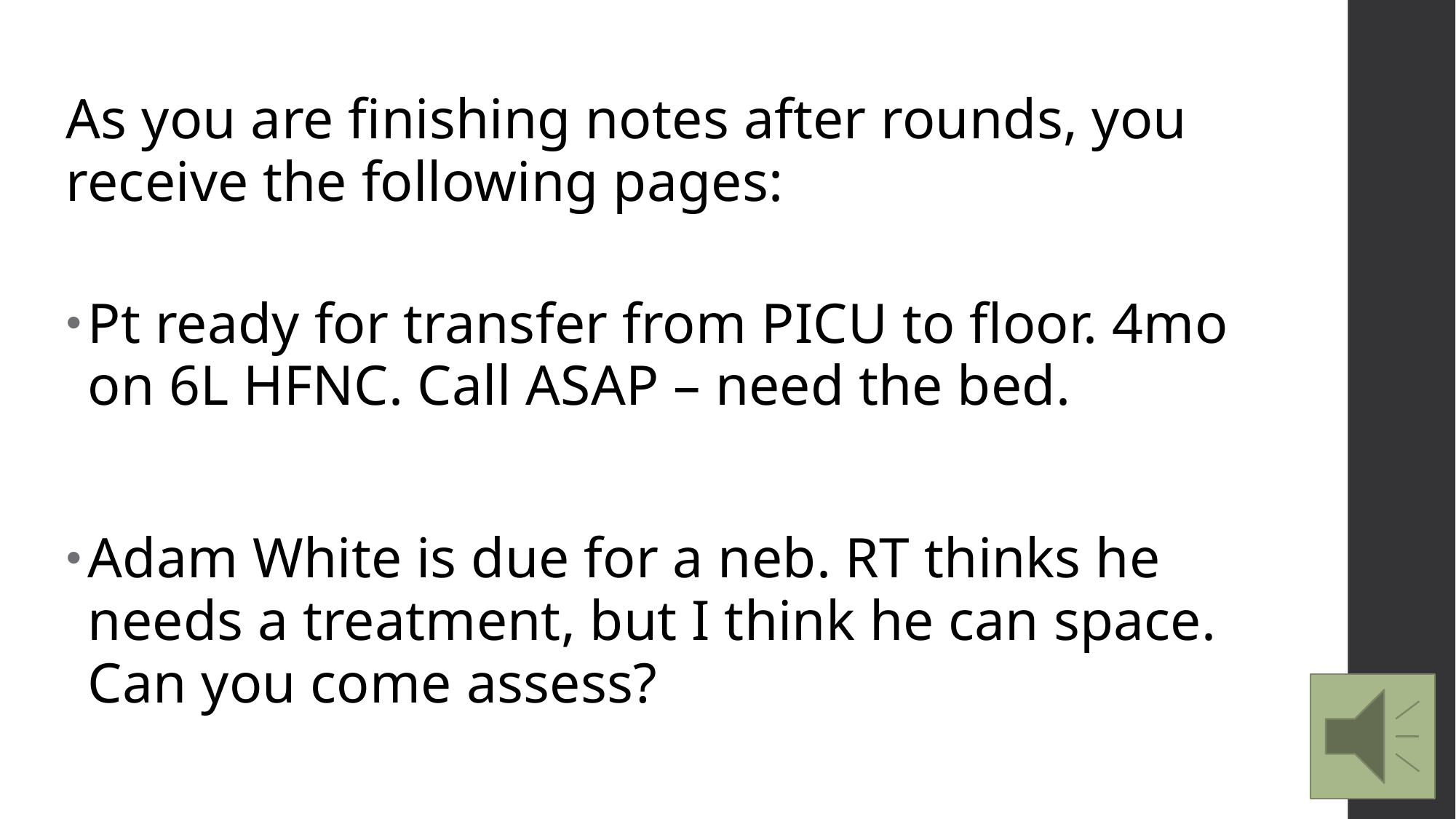

As you are finishing notes after rounds, you receive the following pages:
Pt ready for transfer from PICU to floor. 4mo on 6L HFNC. Call ASAP – need the bed.
Adam White is due for a neb. RT thinks he needs a treatment, but I think he can space. Can you come assess?

## Slide 14
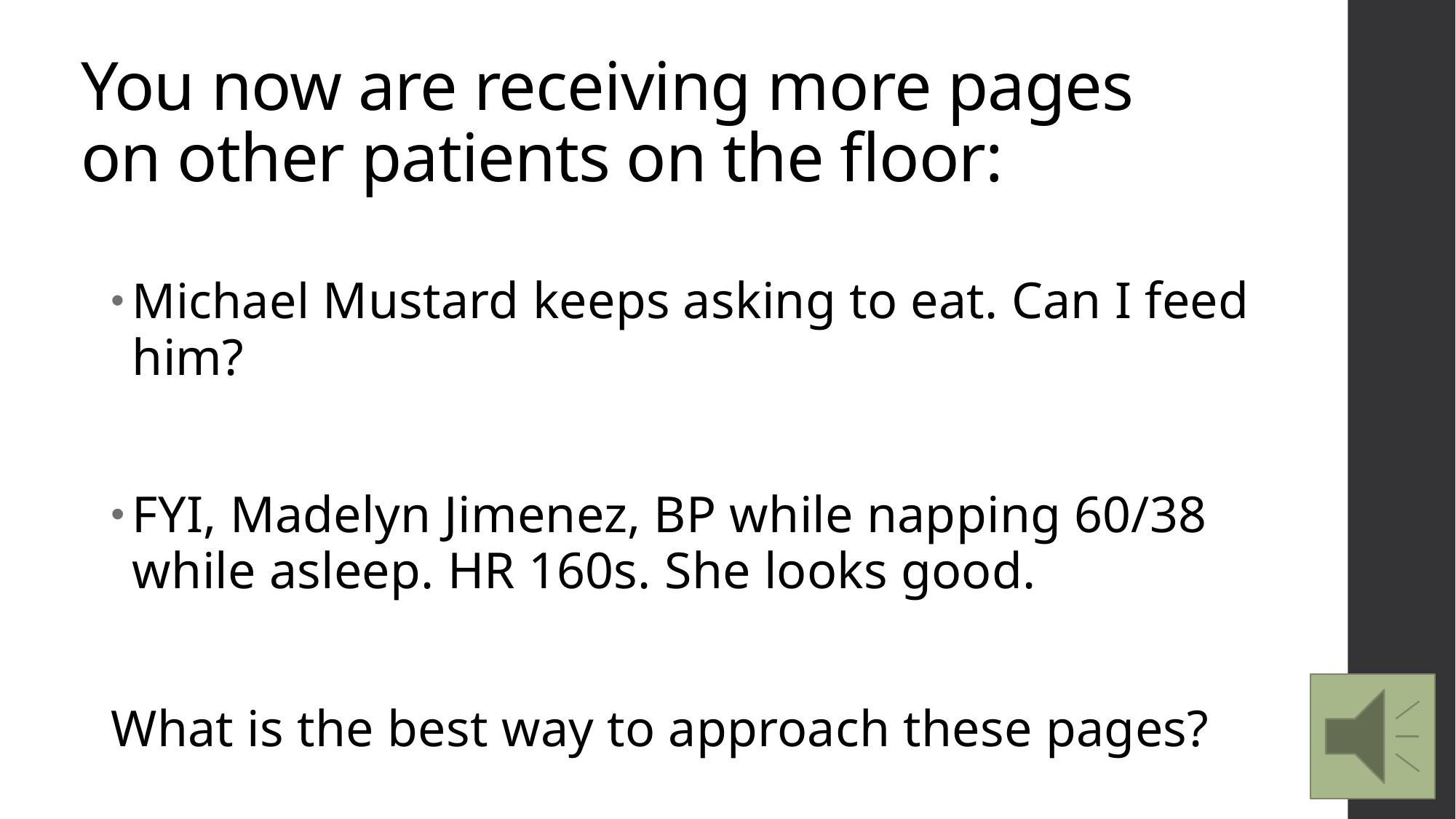

# You now are receiving more pages on other patients on the floor:
Michael Mustard keeps asking to eat. Can I feed him?
FYI, Madelyn Jimenez, BP while napping 60/38 while asleep. HR 160s. She looks good.
What is the best way to approach these pages?

## Slide 15
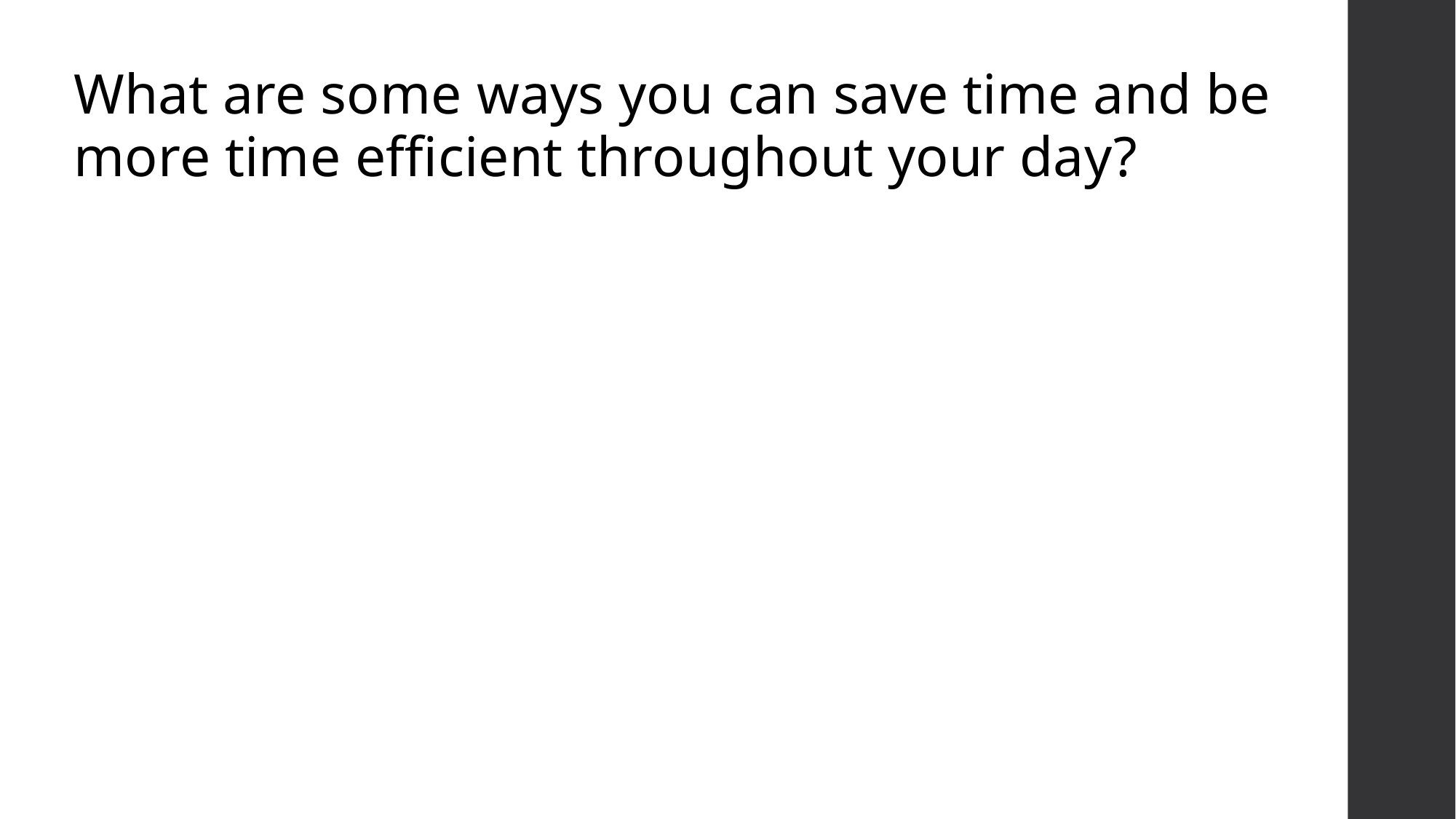

What are some ways you can save time and be more time efficient throughout your day?

## Slide 16
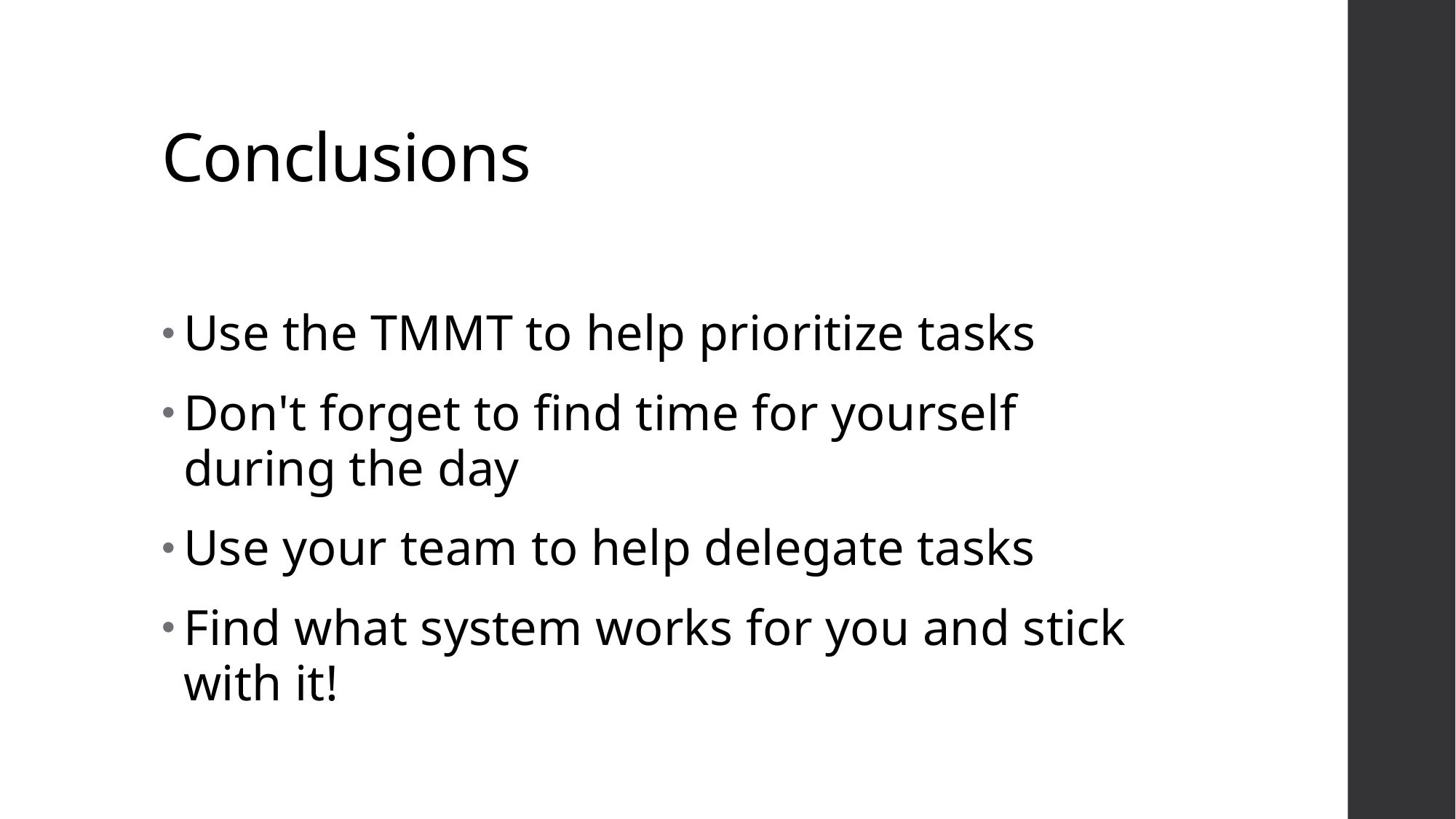

# Conclusions
Use the TMMT to help prioritize tasks
Don't forget to find time for yourself during the day
Use your team to help delegate tasks
Find what system works for you and stick with it!
