# Supplementary material for: Time Management and Task Prioritization Curriculum for Pediatric and Internal Medicine Subinternship Students
Source: MedEdPORTAL. 2022 Feb 22;18:11221. doi: 10.15766/mep_2374-8265.11221 (PMC8861138; doi:10.15766/mep_2374-8265.11221)
Supplement: Supplementary file 1 — Student Survey Evaluations.docxPreworkshop Exercise for Pediatric Students.docxPreworkshop Exercise for Internal Medicine Students.docxWorkshop for Pediatric Students.pptxWorkshop for Internal Medicine Students.pptxSpeaker Notes for Workshop.docx [file mep_2374-8265.11221-s001.zip › E. Workshop for Internal Medicine Students.pptx]

## Slide 1
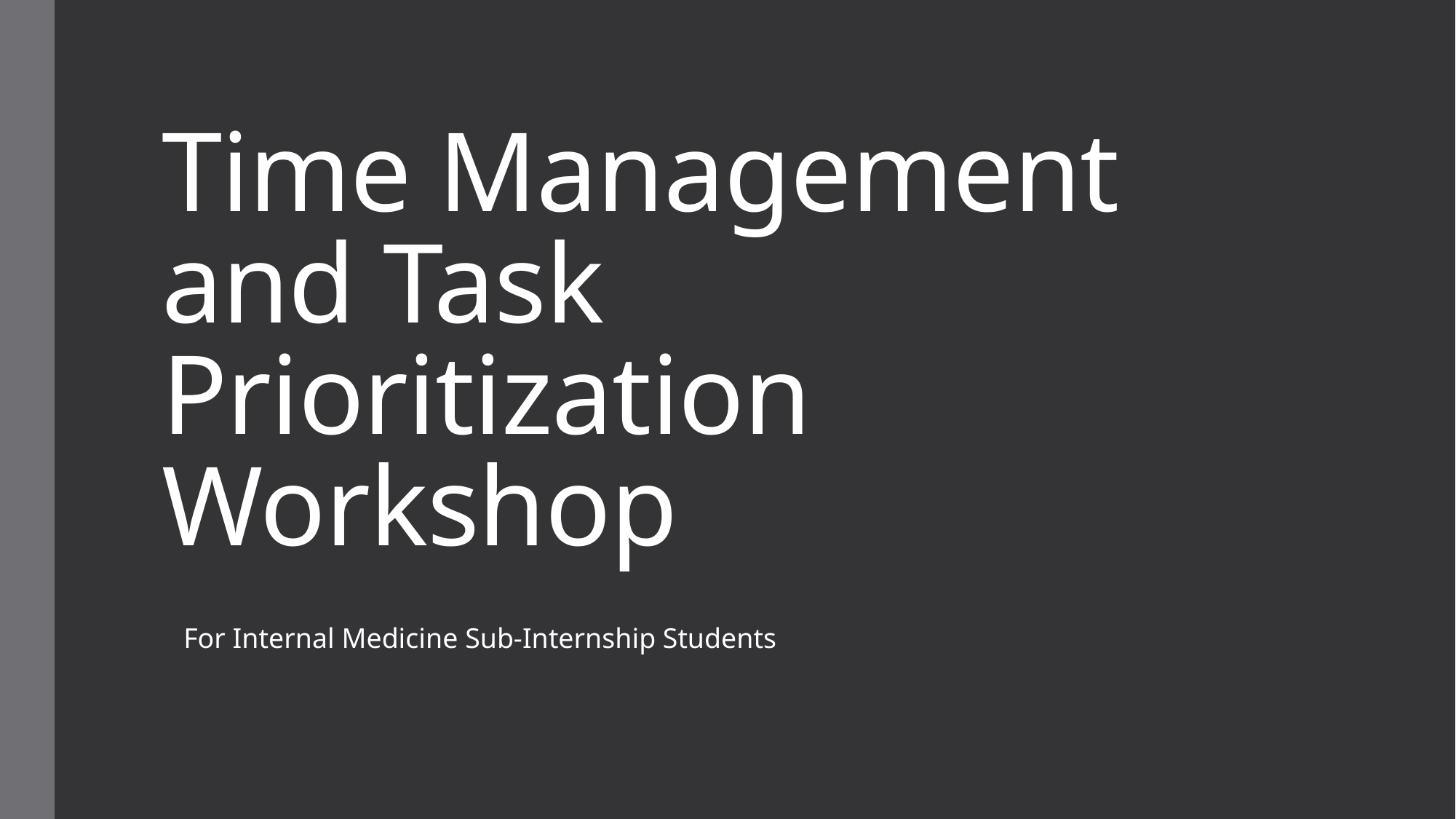

# Time Management and Task Prioritization Workshop
For Internal Medicine Sub-Internship Students

## Slide 2
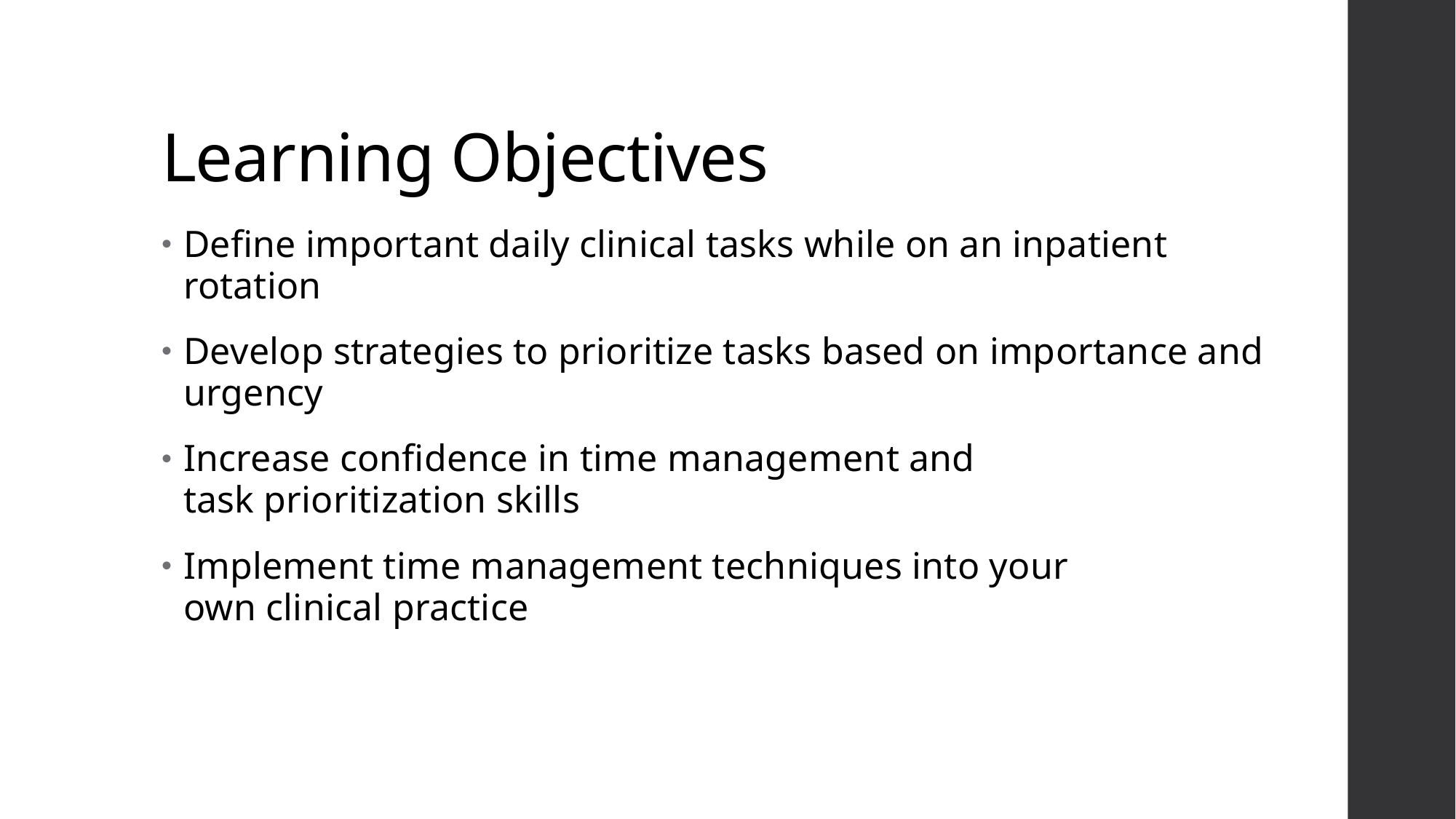

# Learning Objectives
Define important daily clinical tasks while on an inpatient rotation
Develop strategies to prioritize tasks based on importance and urgency
Increase confidence in time management and task prioritization skills
Implement time management techniques into your own clinical practice

## Slide 3
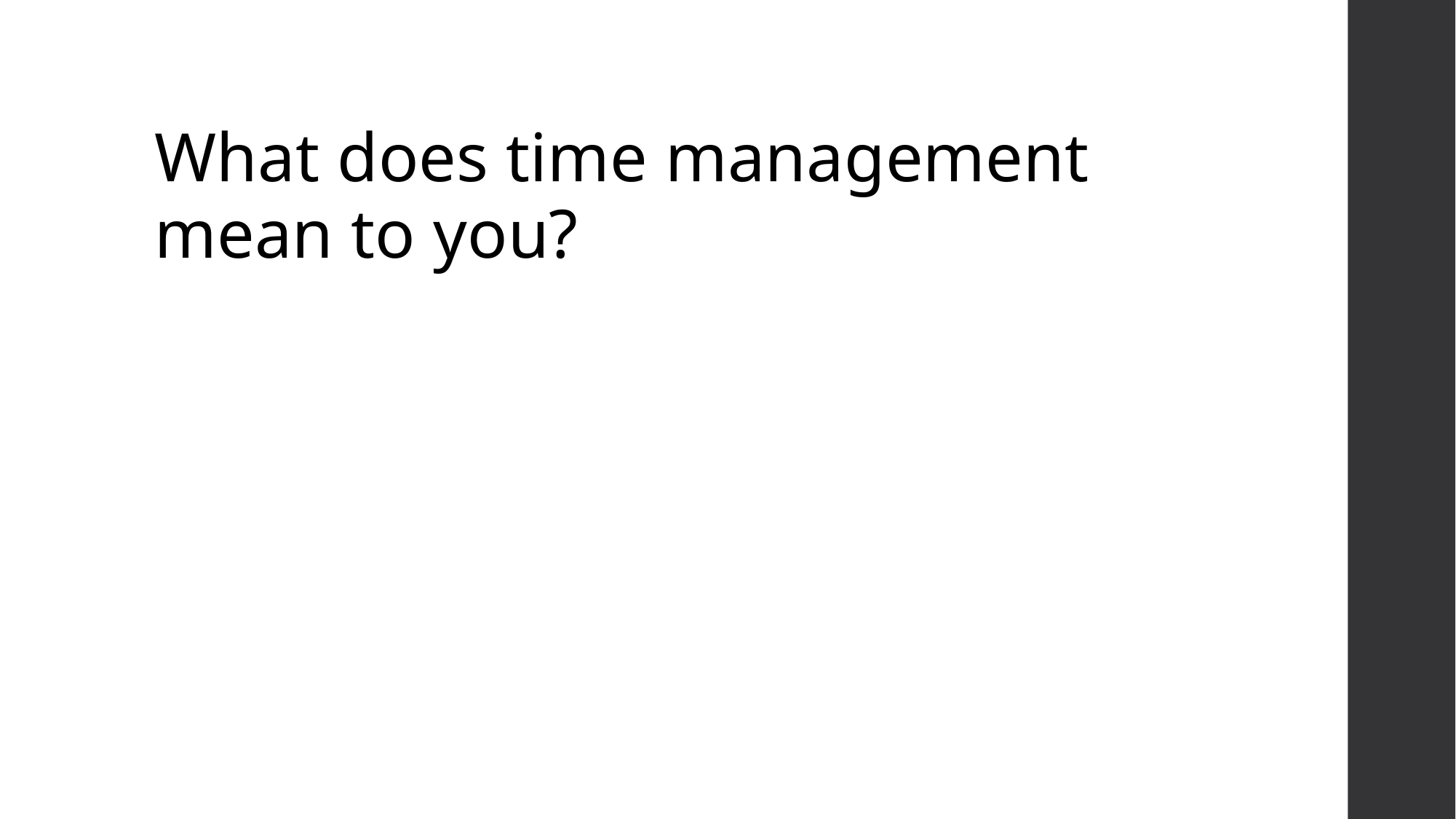

What does time management mean to you?

## Slide 4
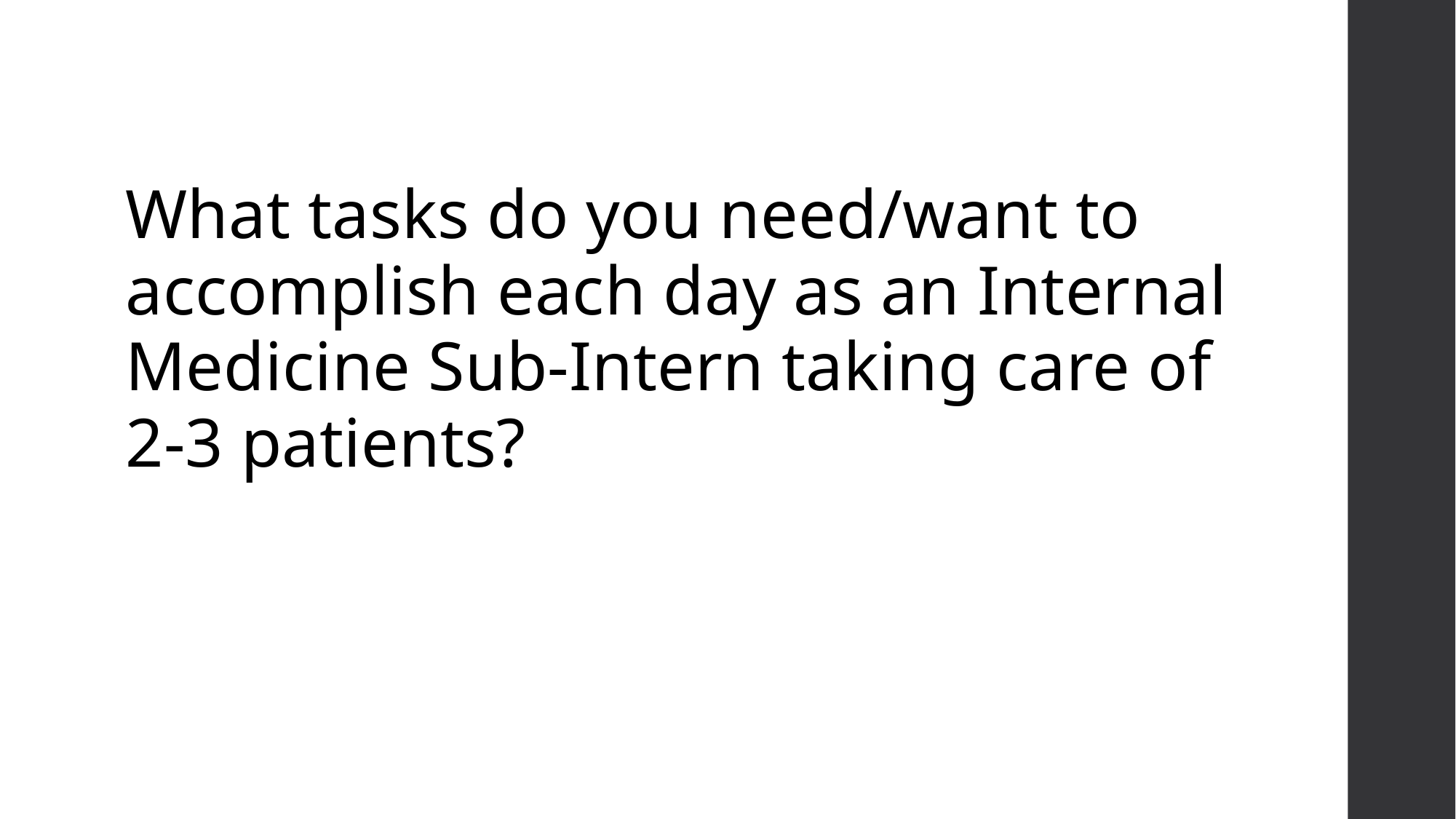

What tasks do you need/want to accomplish each day as an Internal Medicine Sub-Intern taking care of 2-3 patients?

## Slide 5
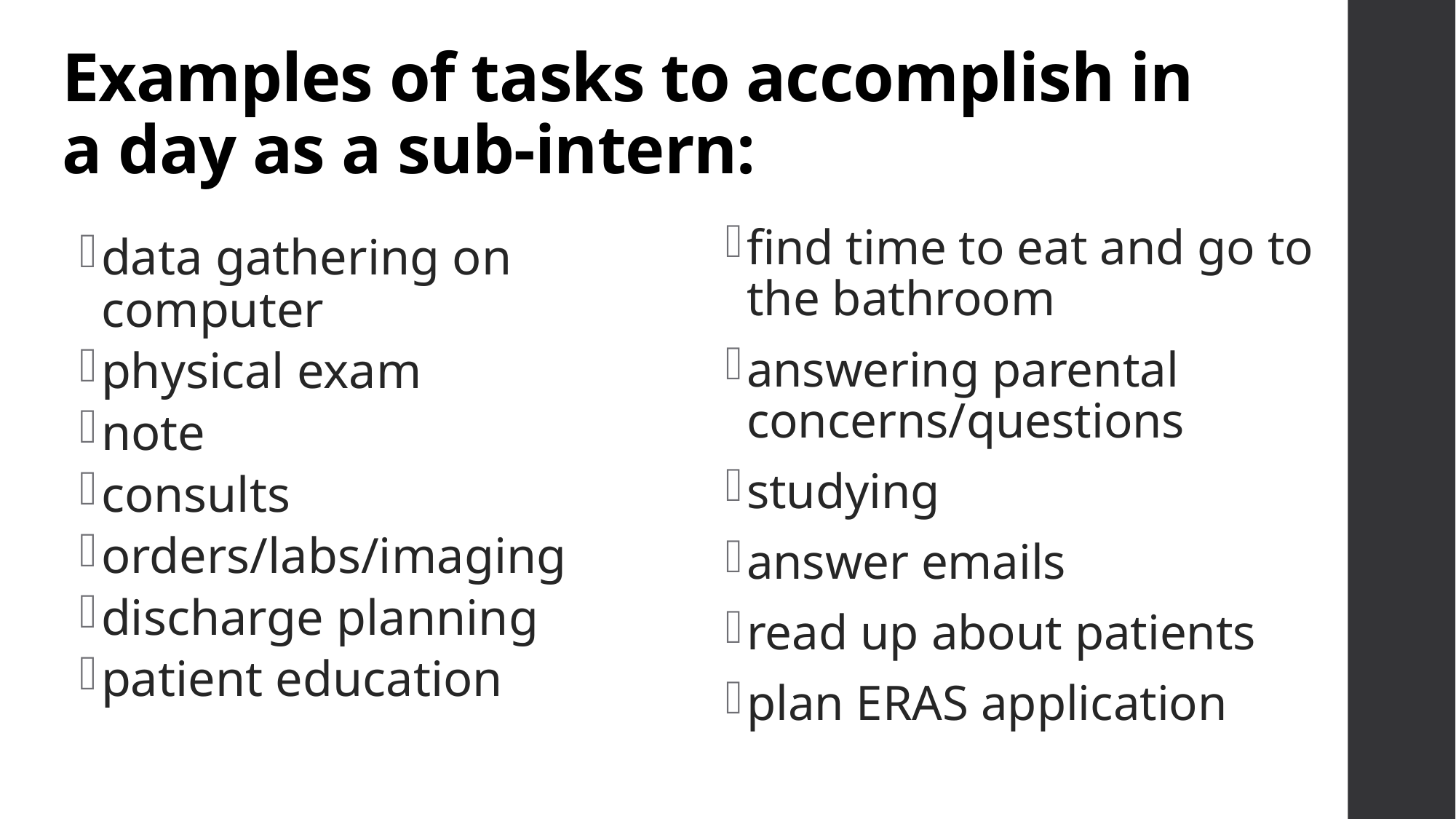

# Examples of tasks to accomplish in a day as a sub-intern:
find time to eat and go to the bathroom
answering parental concerns/questions
studying
answer emails
read up about patients
plan ERAS application
data gathering on computer
physical exam
note
consults
orders/labs/imaging
discharge planning
patient education

## Slide 6
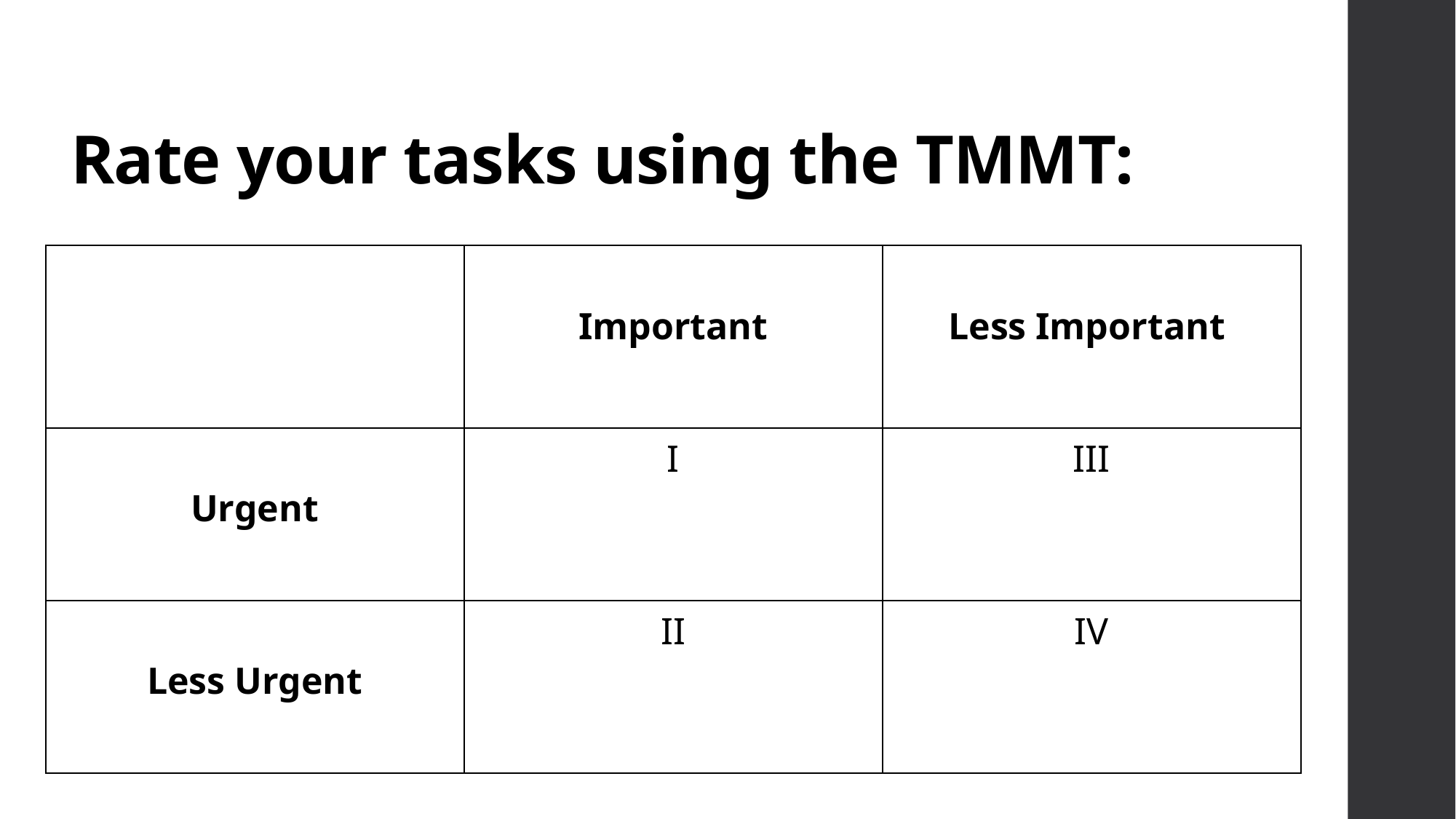

# Rate your tasks using the TMMT:
| | Important | Less Important |
| --- | --- | --- |
| Urgent | I | III |
| Less Urgent | II | IV |

## Slide 7
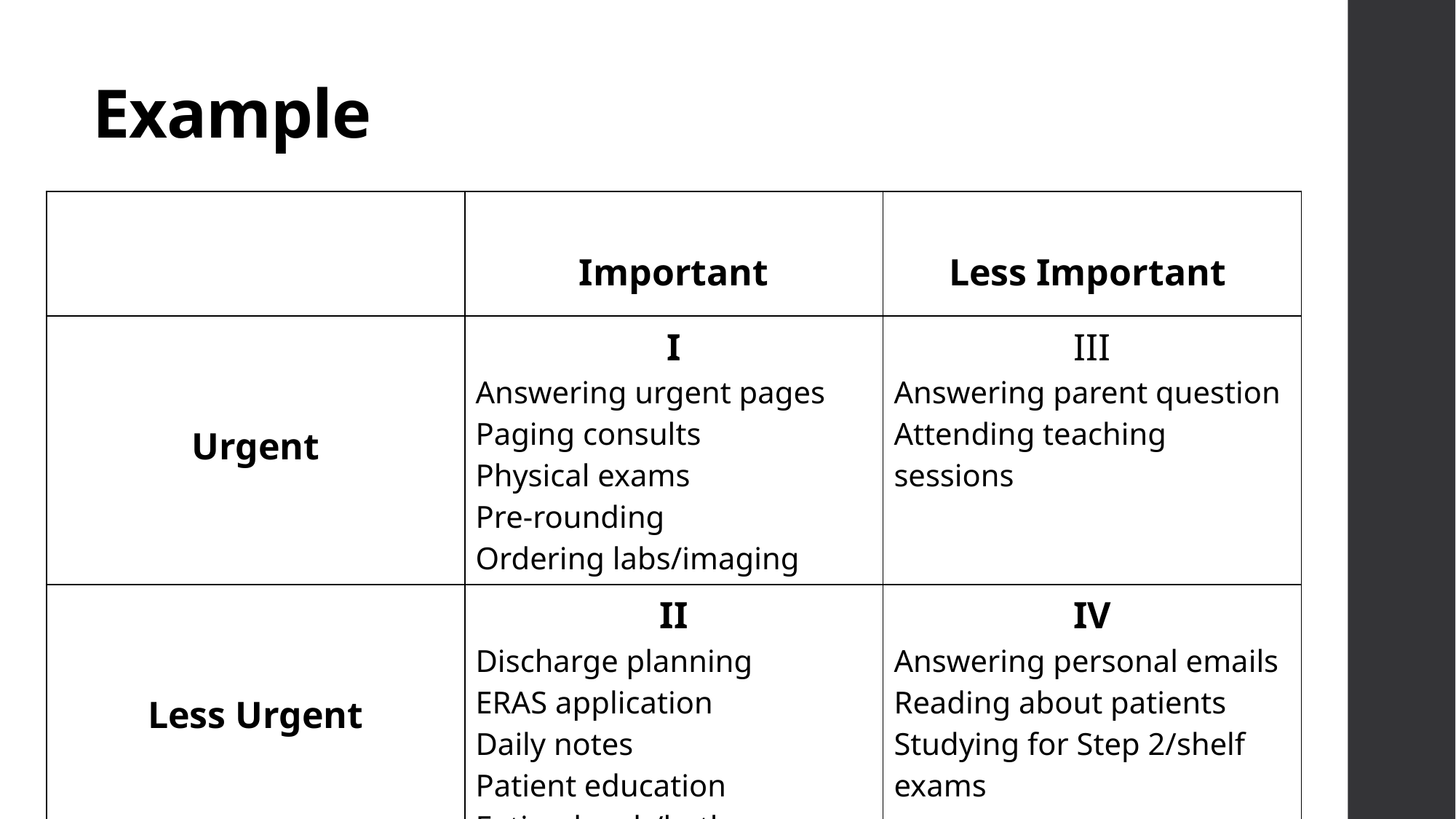

# Example
| | Important | Less Important |
| --- | --- | --- |
| Urgent | I Answering urgent pages  Paging consults Physical exams Pre-rounding Ordering labs/imaging | III Answering parent question Attending teaching sessions |
| Less Urgent | II Discharge planning ERAS application Daily notes Patient education Eating lunch/bathroom | IV Answering personal emails Reading about patients Studying for Step 2/shelf exams |

## Slide 8
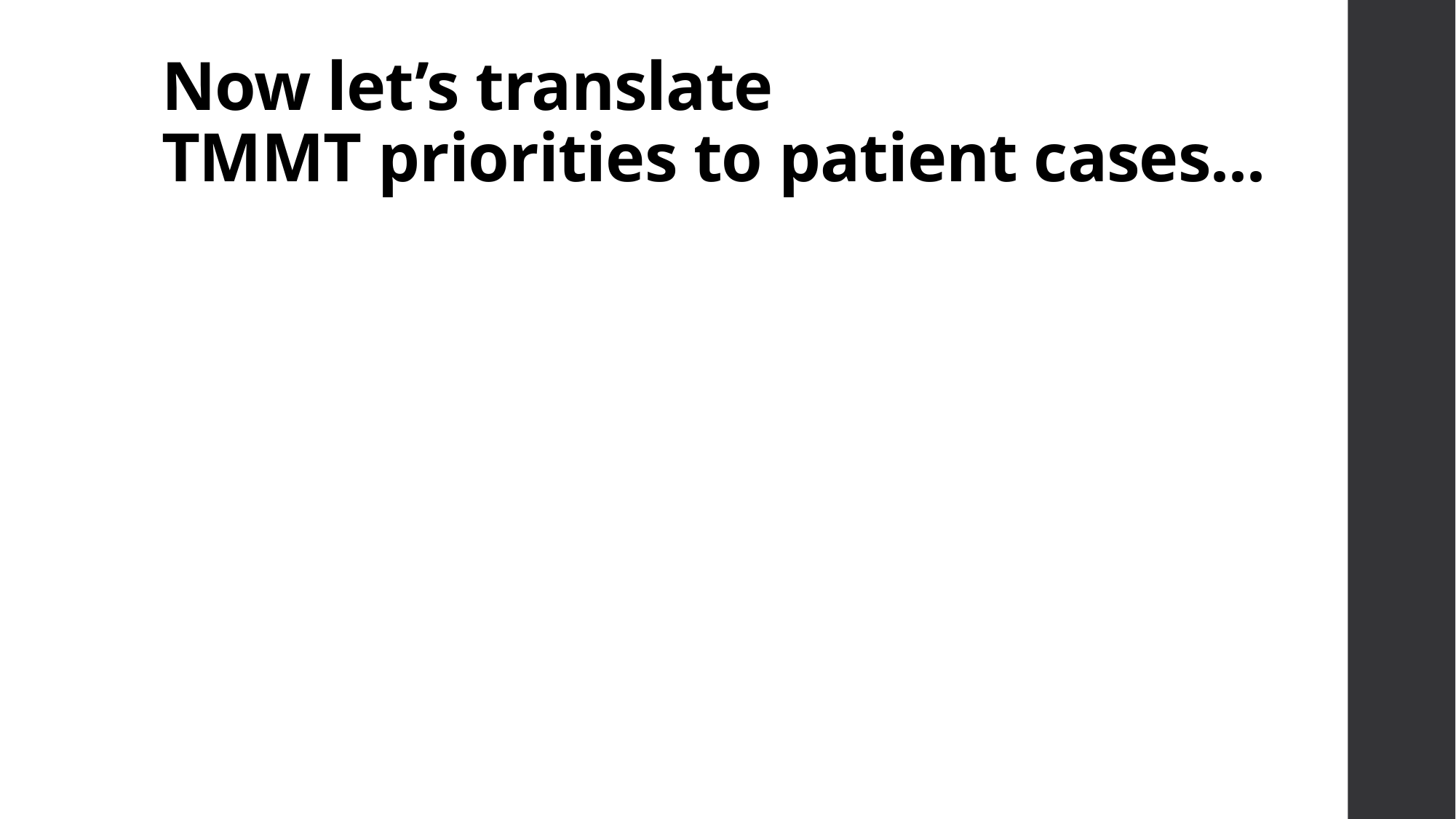

# Now let’s translate TMMT priorities to patient cases...

## Slide 9
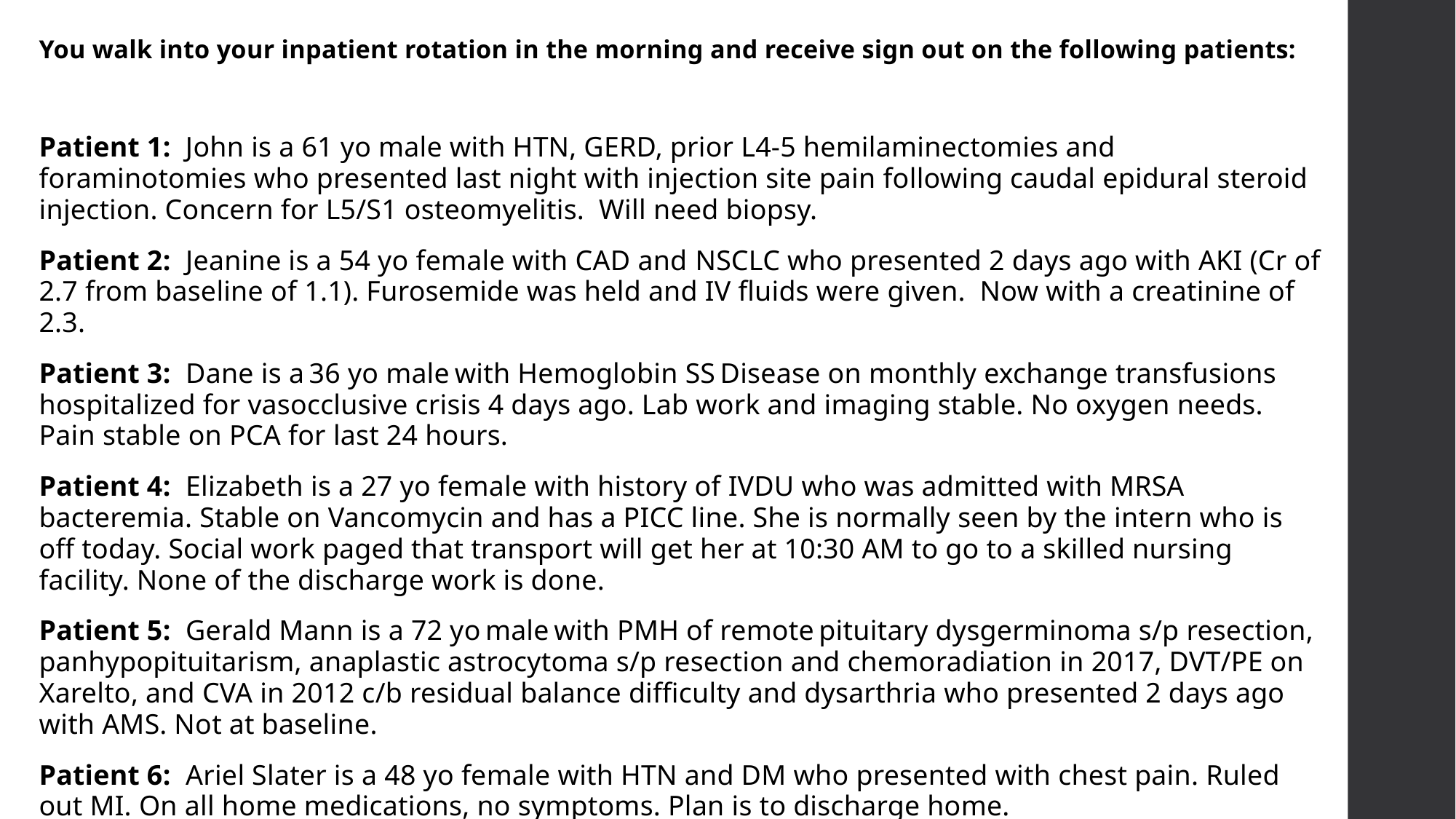

You walk into your inpatient rotation in the morning and receive sign out on the following patients:
Patient 1:  John is a 61 yo male with HTN, GERD, prior L4-5 hemilaminectomies and foraminotomies who presented last night with injection site pain following caudal epidural steroid injection. Concern for L5/S1 osteomyelitis.  Will need biopsy.
Patient 2:  Jeanine is a 54 yo female with CAD and NSCLC who presented 2 days ago with AKI (Cr of 2.7 from baseline of 1.1). Furosemide was held and IV fluids were given.  Now with a creatinine of 2.3.
Patient 3:  Dane is a 36 yo male with Hemoglobin SS Disease on monthly exchange transfusions hospitalized for vasocclusive crisis 4 days ago. Lab work and imaging stable. No oxygen needs. Pain stable on PCA for last 24 hours.
Patient 4:  Elizabeth is a 27 yo female with history of IVDU who was admitted with MRSA bacteremia. Stable on Vancomycin and has a PICC line. She is normally seen by the intern who is off today. Social work paged that transport will get her at 10:30 AM to go to a skilled nursing facility. None of the discharge work is done.
Patient 5:  Gerald Mann is a 72 yo male with PMH of remote pituitary dysgerminoma s/p resection, panhypopituitarism, anaplastic astrocytoma s/p resection and chemoradiation in 2017, DVT/PE on Xarelto, and CVA in 2012 c/b residual balance difficulty and dysarthria who presented 2 days ago with AMS. Not at baseline.
Patient 6:  Ariel Slater is a 48 yo female with HTN and DM who presented with chest pain. Ruled out MI. On all home medications, no symptoms. Plan is to discharge home.

## Slide 10
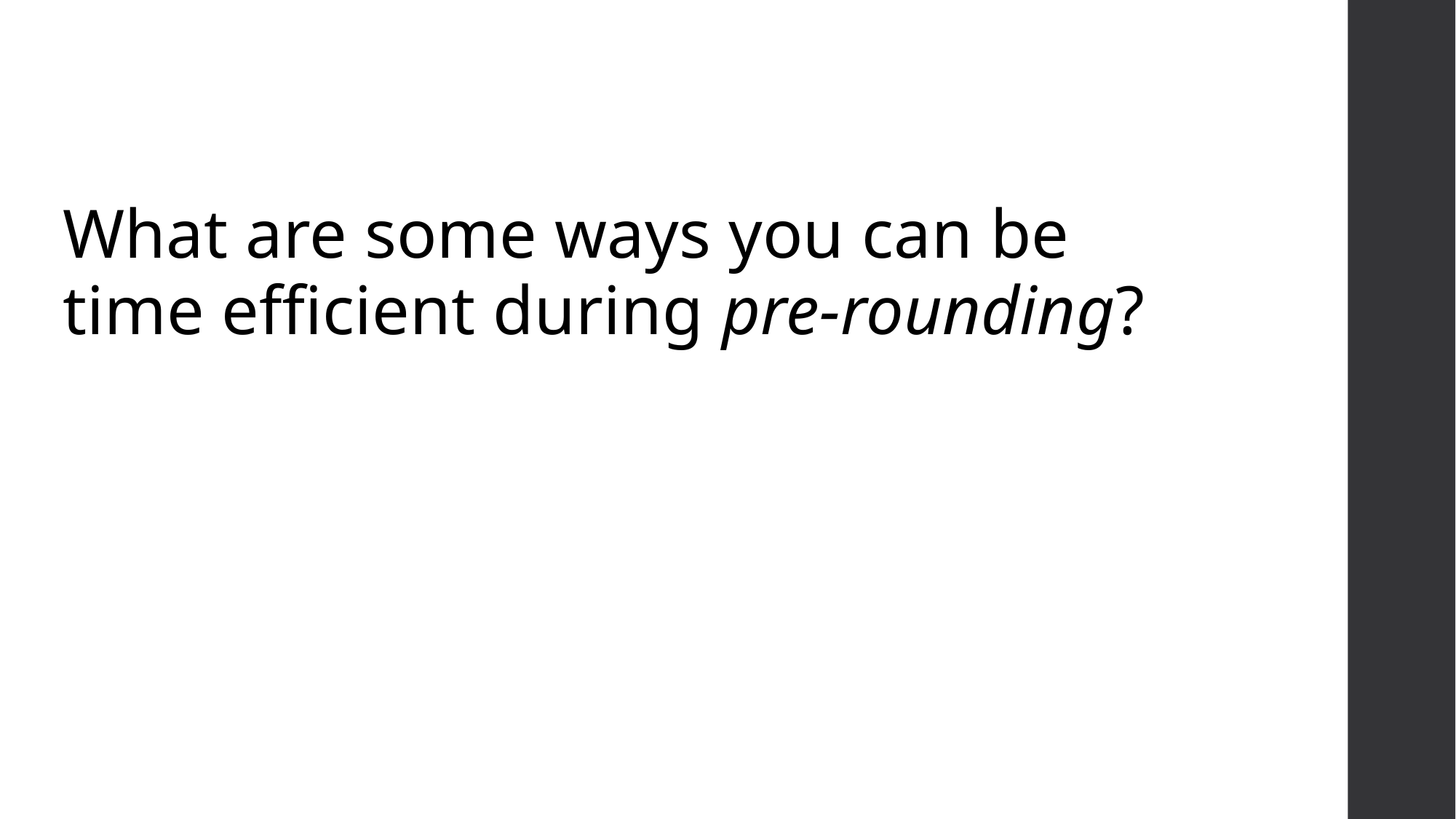

What are some ways you can be time efficient during pre-rounding?

## Slide 11
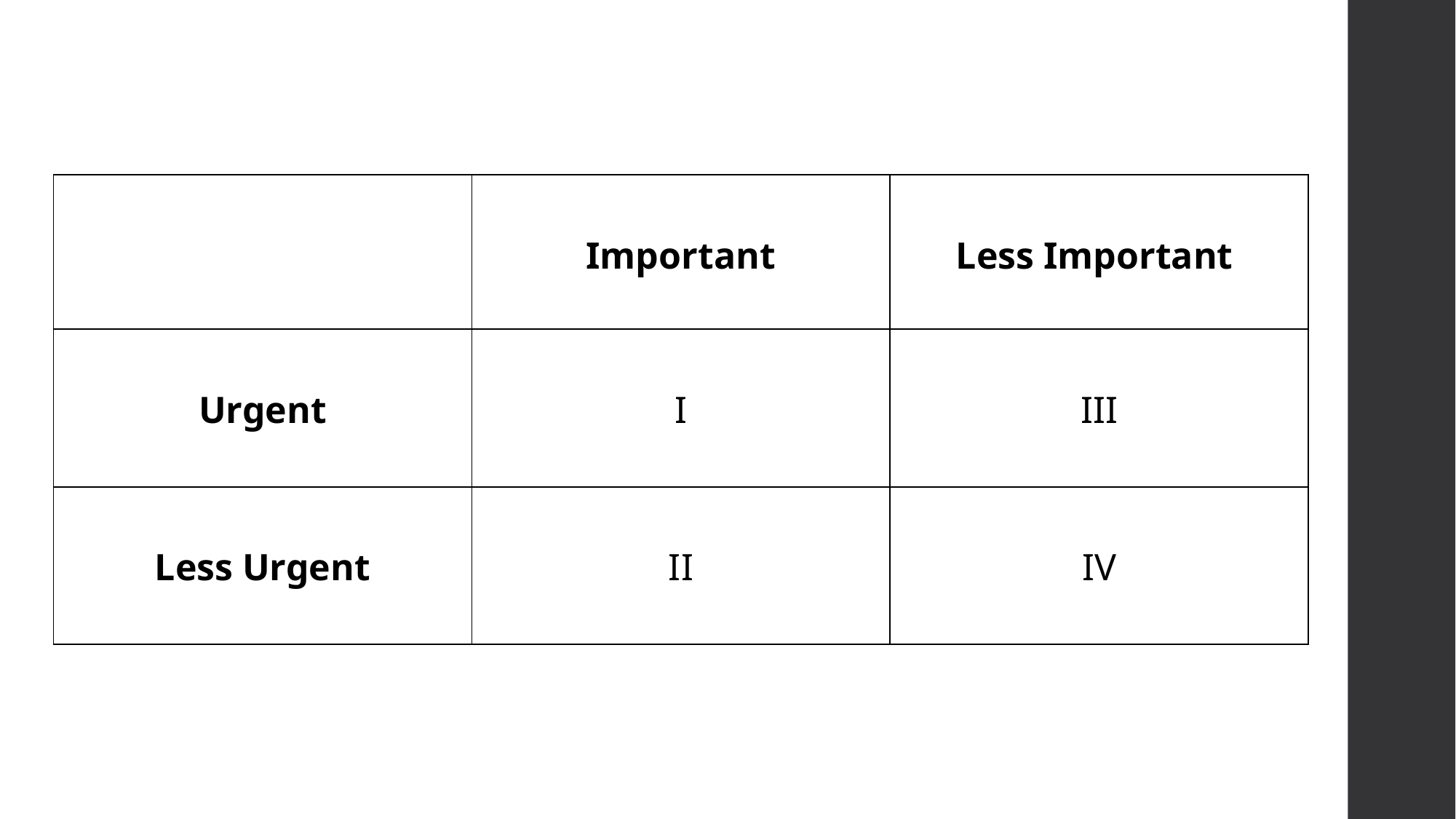

| | Important | Less Important |
| --- | --- | --- |
| Urgent | I | III |
| Less Urgent | II | IV |

## Slide 12
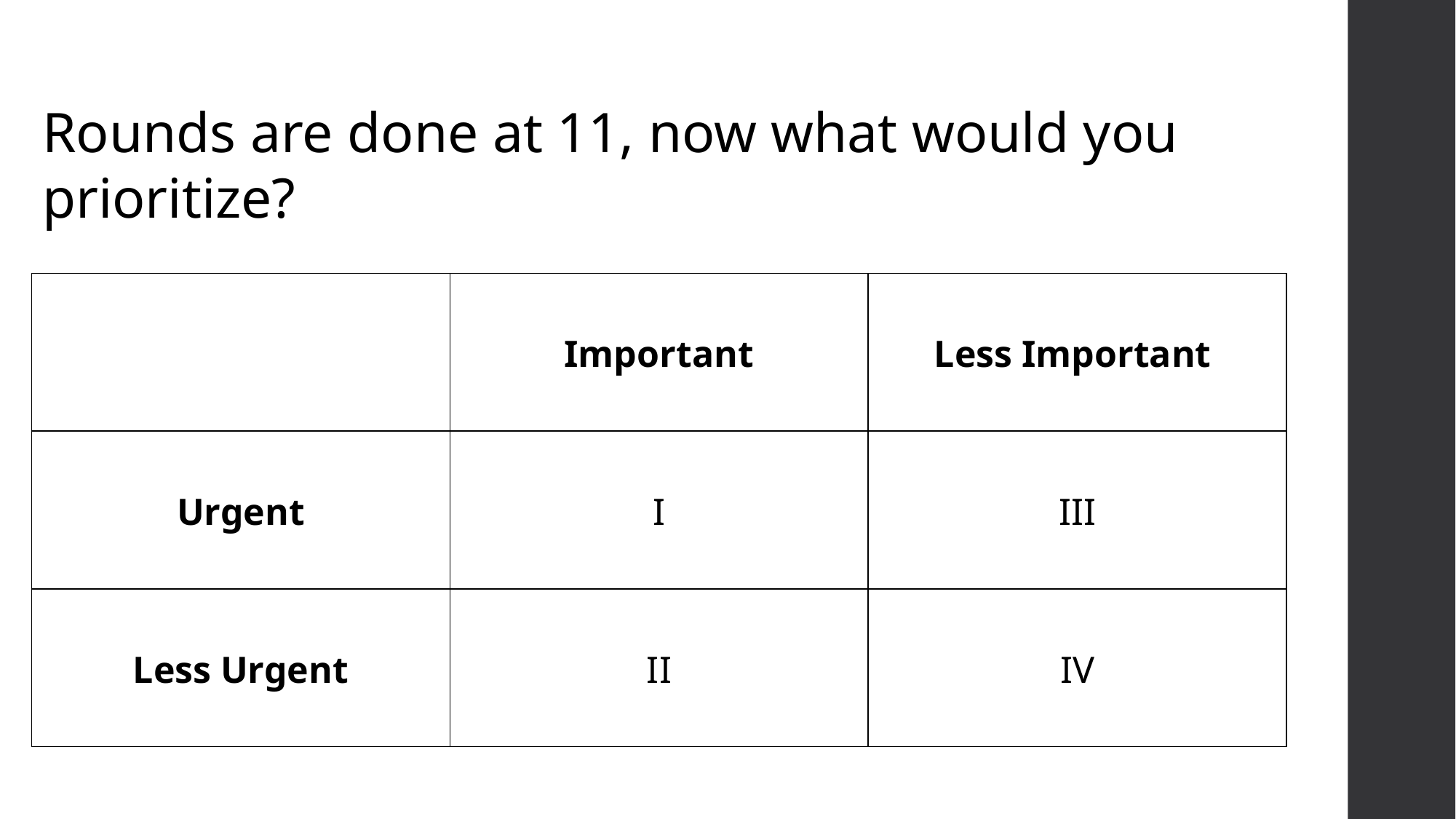

Rounds are done at 11, now what would you prioritize?
| | Important | Less Important |
| --- | --- | --- |
| Urgent | I | III |
| Less Urgent | II | IV |

## Slide 13
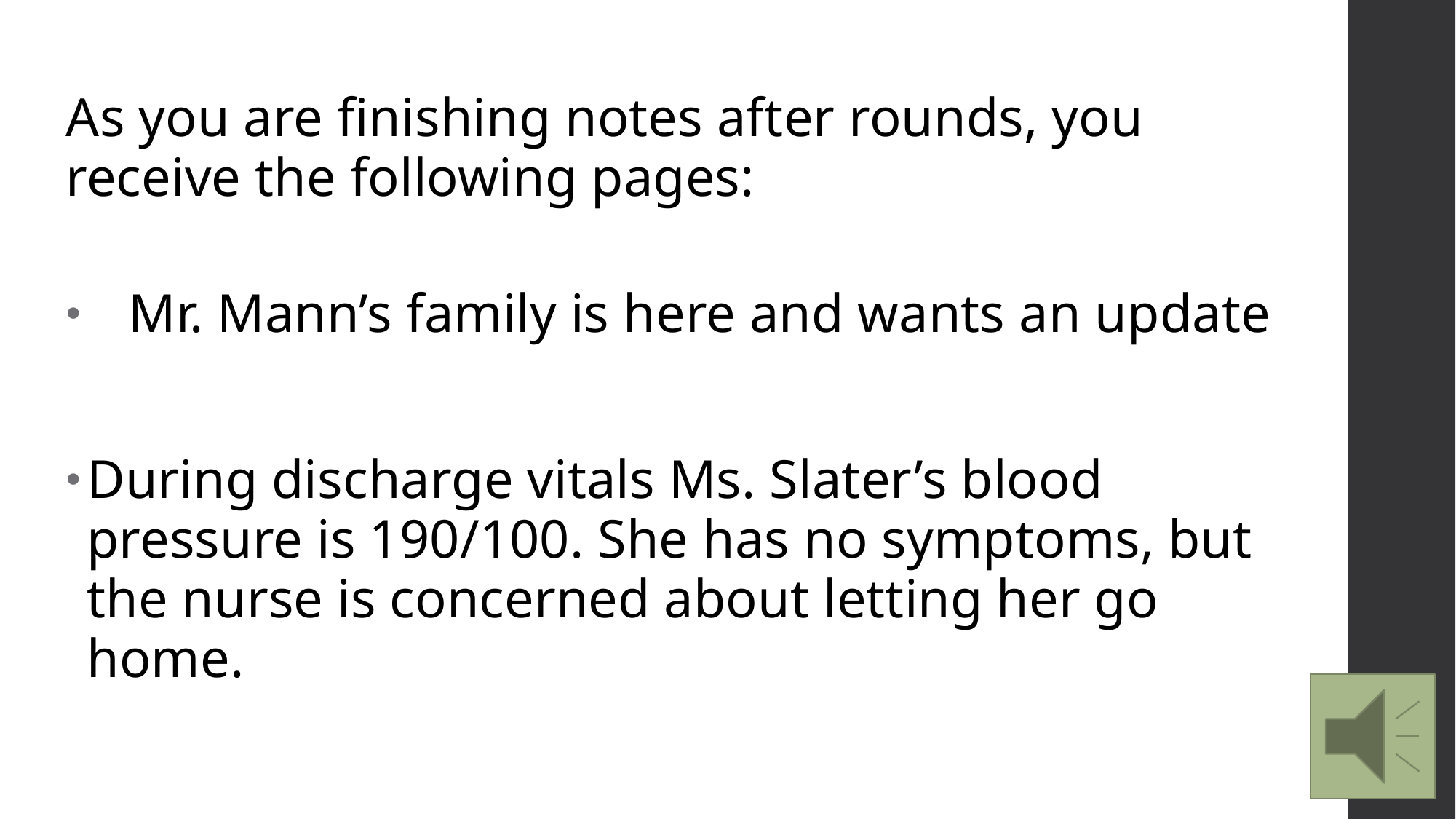

As you are finishing notes after rounds, you receive the following pages:
   Mr. Mann’s family is here and wants an update
During discharge vitals Ms. Slater’s blood pressure is 190/100. She has no symptoms, but the nurse is concerned about letting her go home.

## Slide 14
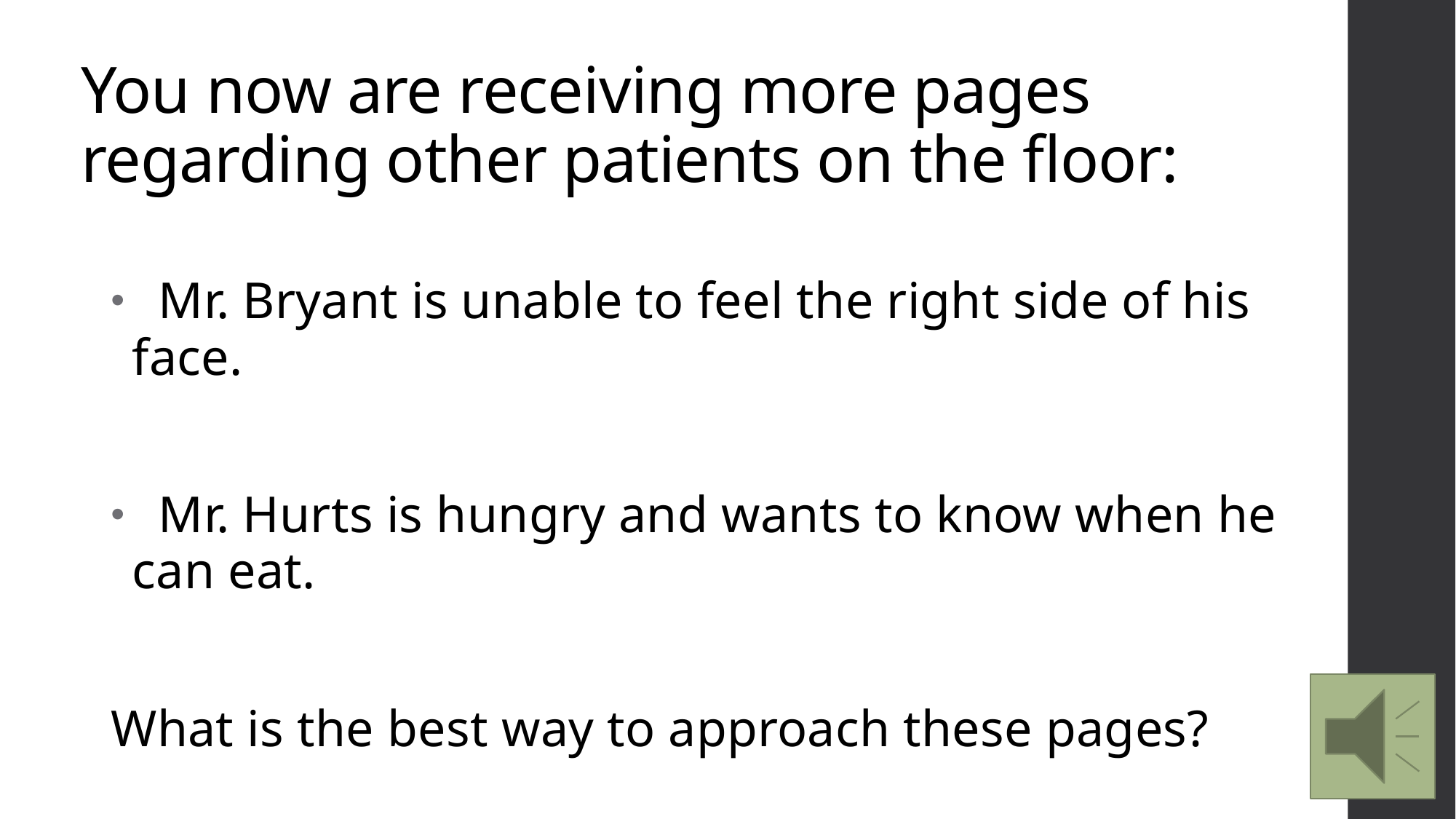

# You now are receiving more pages regarding other patients on the floor:
  Mr. Bryant is unable to feel the right side of his face.
  Mr. Hurts is hungry and wants to know when he can eat.
What is the best way to approach these pages?

## Slide 15
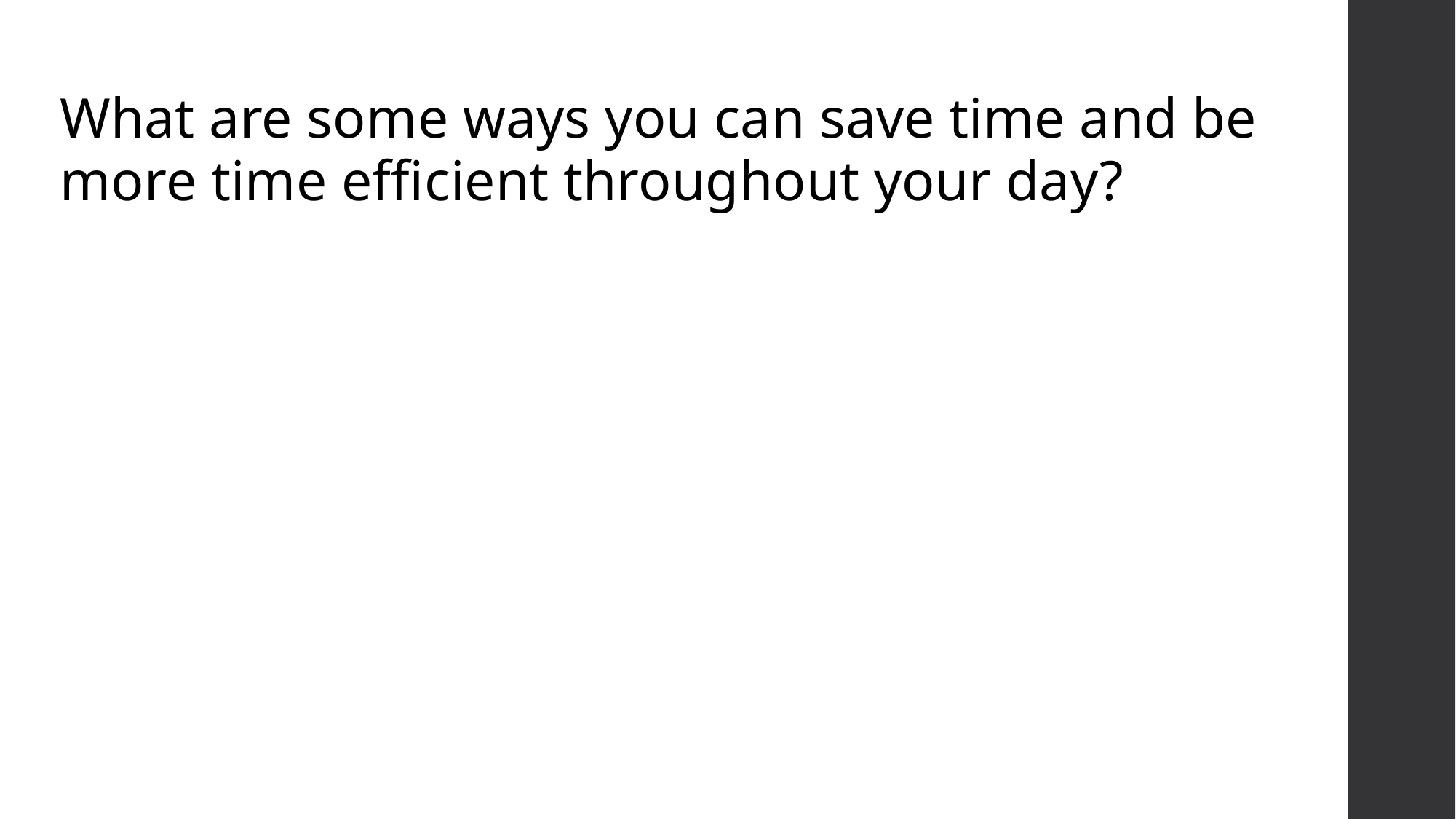

What are some ways you can save time and be more time efficient throughout your day?

## Slide 16
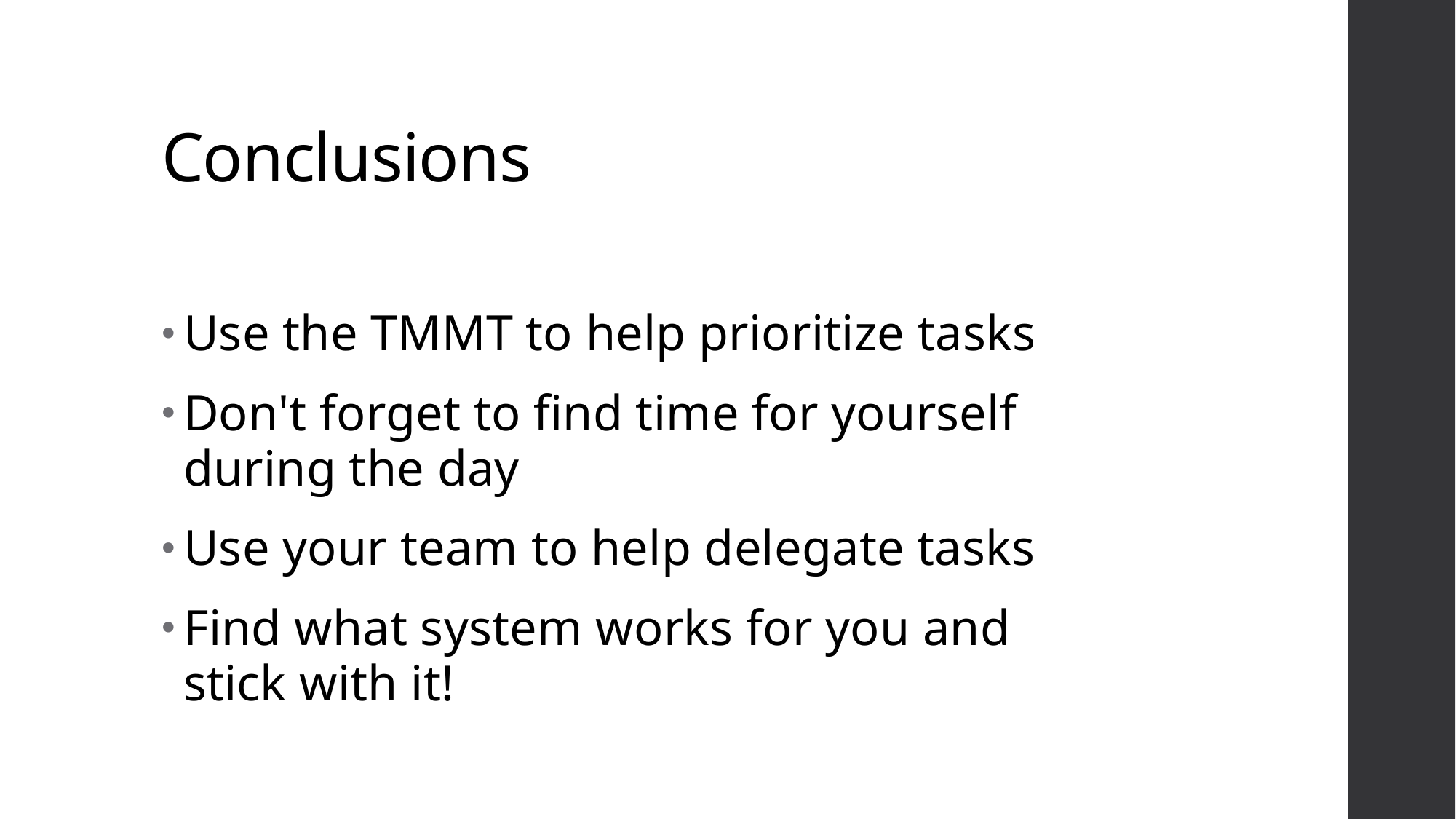

# Conclusions
Use the TMMT to help prioritize tasks
Don't forget to find time for yourself during the day
Use your team to help delegate tasks
Find what system works for you and stick with it!
