# Supplementary material for: Time Management and Task Prioritization Curriculum for Pediatric and Internal Medicine Subinternship Students
Source: MedEdPORTAL. 2022 Feb 22;18:11221. doi: 10.15766/mep_2374-8265.11221 (PMC8861138; doi:10.15766/mep_2374-8265.11221)
Supplement: Supplementary file 1 — Student Survey Evaluations.docxPreworkshop Exercise for Pediatric Students.docxPreworkshop Exercise for Internal Medicine Students.docxWorkshop for Pediatric Students.pptxWorkshop for Internal Medicine Students.pptxSpeaker Notes for Workshop.docx [file mep_2374-8265.11221-s001.zip › F. Speaker Notes for Workshop.docx]

**Speaker Notes for PowerPoint Presentation – for Pediatric Sub-Internship Students**

**Section 1 of Workshop (Refer to slides #1-7)**

**Slide 1:**  The purpose of today's session is to develop time management and task prioritization skills to be used within a clinical setting. In residency, new interns are faced with many challenges associated with their new position. Therefore, developing time management skills early on is important to promote success in residency and your future career.

A portion of today's workshop will be reviewing the pre-work that was assigned a week ago. We will discuss the individual work and complete an additional exercise as a group.

**Slide 2:** By the end of this session, you will be able to define important daily clinical tasks while on an inpatient rotation, develop strategies to prioritize tasks based on importance and urgency, increase confidence in time management and task prioritization skills, and implement time management techniques into your own clinical practice.

**Slide 3:** Time management skills are important within a person's career and balancing work and life priorities. What does time management mean to you?

**Slide 4:** As part of the pre-work exercise, we asked what tasks you need or want to accomplish each day as a pediatric sub-intern taking care of 2-3 patients. Could several students share some of the tasks you listed?

**Slide 5:** Here is an example of several tasks that sub-intern students may need to do within a day while on an inpatient rotation. This includes personal tasks such as finding time to eat, go to the bathroom, studying, answering emails, and starting residency applications.

**Slide 6:** Using the Time Management Matrix Technique or TMMT, rate the importance of each of the tasks you listed in terms of prioritization for the day. For example, what tasks need to happen first and which can wait until later. As a review, the TMMT model is a 4-quadrant table that ranks tasks based on importance and urgency. Many clinical demands are placed in Quadrant I due to level of urgency. Quadrant II are typically activities that require more time and planning but are important for achieving long-term goals and provide career and personal satisfaction.

**Slide 7:** Here is an example of how to prioritize tasks within this model. Quadrant 1 contains tasks related to patients including answering pages, physical exams, placing orders. Quadrant 2 are important tasks but require more time to complete including discharge planning, ERAS/residency applications, writing notes, as well as self-care.

**Section 2 of Workshop (Refer to slides #8-12)**

**Slide 8:** Now let’s translate TMMT priorities to patient cases.

**Slide 9:** You walk into your inpatient rotation in the morning and receive sign out on multiple patients. Of note, these patient cases were included in your presession exercise. However, this slide includes a summary of each patient case. We are going to briefly review them now but please refer to your presession exercise worksheet for additional details.

Patient 1 is Adam, a 5-year-old boy with moderate persistent asthma admitted on every 2-hour albuterol treatments. He was previously hospitalized at an outside hospital, but you do not have the records. He also needs asthma teaching, a follow up PCP appointment and prescriptions prior to discharge.

Patient 2 is Madelyn, a 2 year old female with no significant past medical history who presents with skin lesions concerning for Staphylococcus Scalded Skin Syndrome vs Stevens Johnson Syndrome. The plan today is to consult Dermatology today and collect basic labs. Family speaks only Spanish.

Patient 3 is Jessica, a 2 week female with Trisomy 21 and annular pancreas s/p duodenal duodenostomy, who was transferred from the NICU for feeding management. For the last few days, she has had feeds compressed slowly due to hypoglycemia. No issues overnight with hypoglycemia.

Patient 4 is Abby, a 2yo girl with no significant PMH admitted with pneumonia. She is on 1L of oxygen and IV ampicillin. There are some social concerns regarding home living situation and access to medical care. Plan is to consult Social Work today.

Patient 5 is Rachel, a 2mo ex-34 week gestational age infant with a family history of a sibling who died from SIDS admitted with high-risk BRUE for monitoring. The baby just got admitted at change of shift this morning so the history you received wasn’t the best.

Patient 6 is Michael, a 10 year old boy with no significant PMH admitted with pancreatitis of unclear etiology. Currently NPO with plan to trend labs and obtain GI consult today.

**Slide 10:** After you have received sign-out in the morning, you plan to pre-round on your patients prior to attending rounds. What are some ways you can be time efficient during pre-rounding?

**Slide 11:** Using the TMMT matrix, how will you prioritize the tasks needed for your patients.

Tasks to complete during this time include finishing notes, calling consults, putting in orders, evaluating a particular patient, calling a nurse back, going to rest room, eating a meal/snack, etc.

**Slide 12:** After rounds are done, what tasks do you need to prioritize now?

**Section 3 of Workshop (Refer to slides #12-16)**

**Slide 13:** As you are finishing notes after rounds, you receive the following pages. (*Click on the sound icon then click to reveal the first page on the slide; do the same order prior to revealing the second page*)

- Pt ready for transfer from PICU to floor. 4mo on 6L HFNC. Call ASAP – need the bed.
- Adam White is due for a neb. RT thinks he needs a treatment, but I think he can space. Can you come assess?

How would you prioritize these pages?

**Slide 14:** You now are receiving more pages on other patients on the floor. (*Click on the sound icon then click to reveal the first page on the slide; do the same order prior to revealing the second page*)

- Michael Mustard keeps asking to eat. Can I feed him?
- FYI, Madelyn Jimenez, BP while napping 60/38 while asleep. HR 160s. She looks good.

What is the best way to approach these pages?

**Slide 15:** What are some ways you can save time and be more time efficient throughout your day?

**Slide 16:** In conclusion, you can use the TMMT to help manage your time and prioritize tasks during your clinical rotations. Don’t be afraid to ask your team members for help in delegating tasks and work to find a strategy for time management that works well for you and stick with it.

**Speaker Notes for PowerPoint Presentation – for Internal Medicine Sub-Internship Students**

**Section 1 of Workshop (Refer to slides #1-7)**

**Slide 1:**  The purpose of today's session is to develop time management and task prioritization skills to be used within a clinical setting. In residency, new interns are faced with many challenges associated with their new position. Therefore, developing time management skills early on is important to promote success in residency and your future career.

A portion of today's workshop will be reviewing the pre-work that was assigned a week ago. We will discuss the individual work and complete an additional exercise as a group.

**Slide 2:** By the end of this session, you will be able to define important daily clinical tasks while on an inpatient rotation, develop strategies to prioritize tasks based on importance and urgency, increase confidence in time management and task prioritization skills, and implement time management techniques into your own clinical practice.

**Slide 3:** Time management skills are important within a person's career and balancing work and life priorities. What does time management mean to you?

**Slide 4:** As part of the pre-work exercise, we asked what tasks you need or want to accomplish each day as an internal medicine sub-intern taking care of 2-3 patients. Could several students share some of the tasks placed the list you have created?

**Slide 5:** Here is an example of several tasks that sub-intern students may need to do within a day while on an inpatient rotation. This includes personal tasks such as finding time to eat, go to the bathroom, studying, answering emails, and starting residency applications.

**Slide 6:** Using the Time Management Matrix Technique or TMMT, rate the importance of each of the tasks you listed in terms of prioritization for the day. For example, what tasks need to happen first versus which can wait until later. As a review, the TMMT model is a 4-quadrant table that ranks tasks based on importance and urgency. Many clinical demands are placed in Quadrant I due to level of urgency. Quadrant II are typically activities that require more time and planning but are important for achieving long-term goals.

**Slide 7:** Here is an example of how to prioritize tasks within this model. Quadrant 1 contains tasks related to patients including answering pages, physical exams, placing orders. Quadrant 2 are important tasks but require more time to complete including discharge planning, ERAS/residency applications, writing notes, as well as self-care.

**Section 2 of Workshop (Refer to slides #8-12)**

**Slide 8:** Now let’s translate TMMT priorities to patient cases.

**Slide 9:** You walk into your inpatient rotation in the morning and receive sign out on multiple patients. Of note, these patient cases were included in your presession exercise. However, this slide includes a summary of each patient case. We are going to briefly review them now but please refer to your presession exercise worksheet for additional details.

Patient 1 is John Hurts, a 61 year old man with a past medical history of hypertension, GERD, and prior L4-5 hemilaminectomies and foraminotomies in 2017, who presented to the ED last night with localized injection site pain following caudal epidural steroid injection now with concern for osteomyelitis. Will need biopsy.

Patient 2 is Jeanine Cook, a 54 year old woman with coronary artery disease and non-small cell lung cancer treated in 2012 who presented 2 days ago with AKI (Cr of 2.7 from baseline of 1.1). Furosemide was held and IV fluids were given and now creatinine 2.3.

Patient 3 is Dane Bryant, a 36 year old African American male with history of Hemoglobin SS Disease on monthly exchange transfusions who was hospitalized for vasocclusive crisis 4 days ago. Hgb has been stable. Chest imaging is clear. No oxygen needs. Has been stable on PCA for last 24 hours.

Patient 4 is Elizabeth Clark, a 27 year old female who has a history of IV drug use who was admitted with MRSA bacteremia. She has been stable for last 5 days on Vancomycin and has a PICC line. She is normally seen by the intern who is off today. Social work paged that transport will get her at 10:30 AM to a skilled nursing facility. None of the discharge work (medication reconciliation, discharge summary) has been done.

Patient 5 is Gerald Mann, a 72 year old male with past medical history of remote pituitary dysgerminoma status post resection, panhypopituitarism, anaplastic astrocytoma in the left frontal cortex status post resection and chemoradiation therapy in 2017, DVT/PE on Xarelto, and CVA in 2012 complicated by residual balance difficulty and dysarthria who presented two days ago with altered mental status. Waiting for him to return to baseline.

Patient 6 is Ariel Slater, a 48 year old female with past medical history of hypertension and diabetes who presented with chest pain and has been ruled out for MI and is currently symptom free. Patient is on all her home medications and has no symptoms and the plan is to discharge her.

**Slide 10:** After you have received sign-out in the morning, you plan to pre-round on your patients prior to attending rounds. What are some ways you can be time efficient during pre-rounding?

**Slide 11:** Using the TMMT matrix, how will you prioritize the tasks needed for your patients.
Tasks to complete during this time include finishing notes, calling consults, putting in orders, evaluating a particular patient, calling a nurse back, going to rest room, eating a meal/snack, etc.

**Slide 12:** After rounds are done, what tasks do you need to prioritize now?

**Section 3 of Workshop (Refer to slides #12-16)**

**Slide 13:** As you are finishing notes after rounds, you receive the following pages. (*Click on the sound icon then click to reveal the first page on the slide; do the same order prior to revealing the second page*)

- Mr. Gerald Mann’s family is here and wants an update
- During discharge vitals, Ms. Ariel Slater’s blood pressure is 190/100. She has no symptoms, but the nurse is concerned about letting her go home.

How would you prioritize these pages?

**Slide 14:** You now are receiving more pages on other patients on the floor. (*Click on the sound icon then click to reveal the first page on the slide; do the same order prior to revealing the second page*)

- Mr. Dane Bryant is unable to feel the right side of his face
- Mr. John Hurts is hungry and wants to know when he can eat

What is the best way to approach these pages?

**Slide 15:** What are some ways you can save time and be more time efficient throughout your day?

**Slide 16:** In conclusion, you can use the TMMT to help manage your time and prioritize tasks during your clinical rotations. Don’t be afraid to ask your team members for help in delegating tasks and work to find a strategy for time management that works well for you and stick with it.
