# Supplementary material for: Time Management and Task Prioritization Curriculum for Pediatric and Internal Medicine Subinternship Students
Source: MedEdPORTAL. 2022 Feb 22;18:11221. doi: 10.15766/mep_2374-8265.11221 (PMC8861138; doi:10.15766/mep_2374-8265.11221)
Supplement: Supplementary file 1 — Student Survey Evaluations.docxPreworkshop Exercise for Pediatric Students.docxPreworkshop Exercise for Internal Medicine Students.docxWorkshop for Pediatric Students.pptxWorkshop for Internal Medicine Students.pptxSpeaker Notes for Workshop.docx [file mep_2374-8265.11221-s001.zip › A. Student Survey Evaluations.docx]

**Survey A: Survey provided to students prior to participation in the workshop**

1. In which department will you complete your hospital sub-internship?

Pediatrics Internal Medicine

1. Rate your confidence on your ability to *manage your time effectively* on a clinical rotation.

Very Unconfident Unconfident Neutral Confident Very Confident

1. Rate your confidence on your ability to *prioritize your tasks effectively* on a clinical rotation.

Very Unconfident Unconfident Neutral Confident Very Confident

1. Rate the importance of *time management* for success in residency.

Very Unconfident Unconfident Neutral Confident Very Confident

1. Rate the importance of *task prioritization* for success in residency.

Very Unconfident Unconfident Neutral Confident Very Confident

1. Prior to this rotation have you ever participated in a Time Management/ Tasks Prioritization lecture or workshop before?

Yes No

1. What do you believe is the most beneficial way to learn time management skills prior to residency?
2. I am allowing my data to be used anonymously for research data and publication.

Yes No

**Survey B: Survey provided to students directly after completion of the Time Management Workshop**

1. In which department will you complete your hospital Sub-Internship?

Pediatrics Internal Medicine

1. Do you feel that this workshop will improve your ability to *effectively manage your time* on a clinical rotation?

Strongly Disagree Disagree Neutral Agree Strongly Agree

1. Do you feel that this workshop will improve your ability to *prioritize tasks* on a clinical rotation?

Strongly Disagree Disagree Neutral Agree Strongly Agree

1. Rate your confidence after completing the workshop in your ability to *manage time effectively* on a clinical rotation?

Very Unconfident Unconfident Neutral Confident Very Confident

1. Rate your confidence after completing the workshop in your ability to *prioritize tasks effectively on* a clinical rotation?

Very Unconfident Unconfident Neutral Confident Very Confident

1. Name 2 strategies you learned during this workshop that you plan to use during your sub-internship.
2. This workshop helped me to determine important daily clinical tasks while on my sub-internship.

Strongly Disagree Disagree Neutral Agree Strongly Agree

1. What did this workshop do well?
2. Please provide any suggestions you have to improve the workshop for future students.
