# Supplementary material for: Time Management and Task Prioritization Curriculum for Pediatric and Internal Medicine Subinternship Students
Source: MedEdPORTAL. 2022 Feb 22;18:11221. doi: 10.15766/mep_2374-8265.11221 (PMC8861138; doi:10.15766/mep_2374-8265.11221)
Supplement: Supplementary file 1 — Student Survey Evaluations.docxPreworkshop Exercise for Pediatric Students.docxPreworkshop Exercise for Internal Medicine Students.docxWorkshop for Pediatric Students.pptxWorkshop for Internal Medicine Students.pptxSpeaker Notes for Workshop.docx [file mep_2374-8265.11221-s001.zip › B. Preworkshop Exercise for Pediatric Students.docx]

**Time Management Exercise for Pediatric Sub-Internship Students**

1. **What tasks do you need/want to accomplish each day as a Pediatric Sub-Intern taking care of 2-3 patients?** Think about clinical responsibilities as well as personal responsibilities. For example - finishing notes, calling consults, putting in orders, calling a nurse back, going to rest room, eating a meal/snack, etc.)


1. **Rate the importance of each of the tasks in Question 1 in terms of prioritization for your day. Which one of these needs to happen first, vs which ones can wait until later? Use the Time Management Matrix Technique (TMMT) below.**

|  | **Important** | **Less Important** |
| --- | --- | --- |
| **Urgent** |  |  |
| **Less Urgent** |  |  |

**Now let’s translate your TMMT priorities to patient cases. You arrive to work and receive sign out on several patients. The information you receive on each patient is detailed below. Please read the cases and answer the following questions.**

**Patient 1:** Adam White is a 5 year old boy with moderate persistent asthma who presents with a 2 day history of cough and wheezing. He was hospitalized on the general pediatrics service on every 2 hour albuterol treatments for management of his asthma exacerbation. You want to talk with his nurse and respiratory therapist to see how they think he is doing. He also was hospitalized at an outside hospital PICU about 2 months ago, but you do not have the records and parents are not completely aware of what happened there. He also needs asthma teaching, a follow up PCP appointment and refill prescriptions sent to his pharmacy for when he is ready for discharge.

**Patient 2:** Madelyn Jimenez is a 2 year old female with no significant past medical history who presents with skin lesions concerning for Staphylococcus Scalded Skin Syndrome vs Stevens Johnson Syndrome. Plan is to consult Dermatology today and collect some basic labs (CBC, CMP, CRP, skin cultures). Family speaks only Spanish.

**Patient 3:** Jessica Jones is a 2 week old female with Trisomy 21 and annular pancreas status post duodenal duodenostomy who was transferred from the NICU to the pediatric floor for feeding management. For the last few days, she has had feeds compressed slowly due to hypoglycemia. She is making progress and tolerating feeds well. There were no issues with hypoglycemia overnight.

**Patient 4:** Abby Peacock is a 2 year old girl with no significant past medical history who was admitted with pneumonia. She is currently on 1 L of oxygen via nasal cannula and IV ampicillin. There are some social concerns regarding home living situation and access to medical care. The plan is to consult Social Work today.

**Patient 5:** Rachel Carter is a 2 month old female born at 34-weeks gestation with a family history of a sibling who died from SIDS. Rachel is currently admitted for monitoring after a high-risk BRUE. The baby got admitted at change of shift so the history you received wasn’t the best.

**Patient 6:** Michael Mustard is a 10 year old boy with no significant past medical history admitted with pancreatitis of unclear etiology. Currently NPO with plan to trend labs and obtain GI consult.

**You have received sign-out on your patients in the morning and are planning your time before attending rounds. What are some ways you can be time efficient during *pre-rounding*?**


**You have finished rounding on your patients in the morning. You are now trying to think about what you could do before attending rounds start in the next 30 minutes. Tasks can include finishing notes, calling consults, putting in orders, evaluating a particular patient, calling a nurse back, going to rest room, eating a meal/snack, etc. Use the TMMT matrix.**

|  | **Important** | **Less Important** |
| --- | --- | --- |
| **Urgent** |  |  |
| **Less Urgent** |  |  |

**Rounds are done at 11 AM, now what would you prioritize?**
