# Supplementary material for: Time Management and Task Prioritization Curriculum for Pediatric and Internal Medicine Subinternship Students
Source: MedEdPORTAL. 2022 Feb 22;18:11221. doi: 10.15766/mep_2374-8265.11221 (PMC8861138; doi:10.15766/mep_2374-8265.11221)
Supplement: Supplementary file 1 — Student Survey Evaluations.docxPreworkshop Exercise for Pediatric Students.docxPreworkshop Exercise for Internal Medicine Students.docxWorkshop for Pediatric Students.pptxWorkshop for Internal Medicine Students.pptxSpeaker Notes for Workshop.docx [file mep_2374-8265.11221-s001.zip › Educational_Summary_Report_11221.pdf]

MedEdPORTAL® The AAMC Journal of Teaching and Learning Resources

Original Publication Open Access

Time Management and Task Prioritization Curriculum for Pediatric and Internal Medicine Subinternship Students

Megan Murphy, MD*, Amit Pahwa, MD, Barbara Dietrick, MD, Nicole Shilkofski, MD, MEd, Carly Blatt, MD

*Corresponding author: mmurp101@jhmi.edu

Abstract

Introduction: As a physician, it is important to develop time management and task prioritization skills early to promote future career success. In medical education, there is minimal structured time to teach these skills prior to residency. Stephen Covey's Time Management Matrix Technique (TMMT) is one strategy that can be used to develop these skills. This technique categorizes tasks into a four-quadrant table based on importance and urgency. Using this technique as a model, the authors developed a workshop for medical students on an inpatient pediatric or internal medicine subinternship. **Methods:** Prior to the workshop, students read an article and completed a survey and two self-directed exercises. The exercises asked students to create a list of tasks, develop an individualized TMMT model, and review specialty-specific patient cases. The workshop consisted of discussions on the presession work and group exercises on prioritizing tasks and responding to patient-related pages. Students evaluated the curriculum after the workshop with a survey. **Results:** Most participants (82%) strongly agreed or agreed that the workshop improved their ability to manage time effectively and prioritize tasks on a clinical rotation. There was a statistically significant increase in both median time management and task prioritization confidence scores after completion of the workshop ($p < .05$). **Discussion:** This workshop provides one strategy that can be implemented within undergraduate medical education to enhance time management skills prior to residency. Future studies should be aimed at evaluating these skills within the clinical setting.

Keywords
Time Management, Curriculum Development, Case-Based Learning, Clinical Skills Training

Educational Objectives

By the end of this workshop, learners will be able to:

1. Define important daily clinical tasks while on a subinternship rotation.
2. Acquire the skills needed to prioritize tasks using the Time Management Matrix Technique.
3. Report increased confidence in development of time management and task prioritization skills.
4. Implement time management techniques into their own clinical practice during their subinternship.

Citation:
Murphy M, Pahwa A, Dietrick B, Shilkofski N, Blatt C. Time management and task prioritization curriculum for pediatric and internal medicine subinternship students. *MedEdPORTAL.* 2022;18:11221. https://doi.org/10.15766/mep_2374-8265.11221

Introduction

As a physician, it is essential to learn how to improve efficiency and develop appropriate time management skills to promote future career success.[1] Implementing these skills early in a person's career is also important to prevent burnout. After graduating from medical school, a first-year postgraduate resident is faced with many challenges, including balancing the numerous demands of their new profession. Unfortunately, there is limited structured teaching on time management skills during medical education. A survey conducted by Susan Miles and colleagues in 2017 revealed that task prioritization and time management were both considered problematic areas for new doctors.[2] Physicians have also reported increasing burnout rates in every specialty attributable to "spending too many hours at work" and "too many bureaucratic tasks," which are both related to time management.[3] In recent years, there has been an increased emphasis on the importance of work-life balance among medical professionals. Maximizing time outside of work is directly related to individual efficiency and prioritization of tasks.

Copyright © 2022 Murphy et al. This is an open-access publication distributed under the terms of the Creative Commons Attribution license.

There are descriptions of previous workshops that were developed to teach time management skills. These workshops included a significant didactic component and were aimed at participants who were physicians early on in their careers, such as residents, fellows, and junior faculty.[4] Even though workshops have described the importance of additional training early in an individual's career, there has been little focus on enhancing the skills of time management in medical school to prepare students for residency. There is also a lack of evidence regarding the best mechanism for teaching these skills, whether didactic presentations, self-directed learning, or group discussion. Thus, we identified a need for developing a curriculum that would be interactive and effective in enhancing these skills within medical students.

Stephen R. Covey's Time Management Matrix Technique (TMMT) is one strategy that can be used when developing and improving upon time management skills.[1,5,6] This technique uses a four-quadrant table to categorize tasks based on importance and urgency. By visualizing the matrix, a physician can assess how much time is spent completing activities in each category and organize their priorities accordingly.[7] Figure 1 shows an example of a TMMT model with tasks that a physician may encounter daily and how they can prioritize these tasks based on importance and urgency for completion. Based on the TMMT and incorporating David Kolb's experiential learning cycle framework, we created a time management workshop specifically aimed at pediatric and internal medicine subinternship students.[8,9]

	Important	Less Important
Urgent	I<br>Answering urgent pages about patients.<br>Physical examinations.<br>Ordering labs and imaging.	III<br>Answering parent questions.<br>Attending teaching sessions.
Less Urgent	II<br>Discharge planning.<br>Daily notes.	IV<br>Answering personal emails.<br>Reading about patients.<br>Studying for board examinations.

Figure 1. Covey's Time Management Matrix Technique. The four quadrants allow for categorization of tasks based on importance and urgency. Each quadrant provides a list of daily tasks a physician may have and an example of how those tasks may be ranked in order of importance and urgency.

The goal of this workshop is to enhance time management and task prioritization skills prior to residency and provide strategies that can be implemented in clinical practice. We utilize a combination of didactic teaching, group discussion, and role-play. Students are initially given active prework didactics to introduce the concept of the TMMT and time management strategies. Within the live workshop, students participate in group discussions to share their thoughts. Lastly, participants delve into role-playing to actively experiment with the material in accordance with Kolb's theories.[9] All three strategies require active learning and participation on behalf of the participant to acquire the skills of time management and then apply their knowledge to real cases.

Methods

We designed the curriculum for fourth-year medical students rotating on an inpatient unit for a pediatric or internal medicine subinternship. There were five total iterations of the workshop completed throughout the year. The workshop was held prior to the start of the students' subinternship rotation, with four to 10 students present at each iteration. To participate in this workshop, students were required to be enrolled in, but not yet have completed, a pediatrics or internal medicine subinternship rotation. Additionally, as a prerequisite to the live workshop session, students were required to complete preworkshop activities, as listed under the Preworkshop Self-Directed Learning section below. During the workshop, there were one to two facilitators present to lead the exercises and encourage student discussions. Facilitators were either resident or attending physicians in pediatrics or internal medicine. Facilitators were also required to complete the preworkshop activities listed below in order to assist in the discussions for the workshop.

Preworkshop Self-Directed Learning

Students were required to complete a survey (Appendix A: Survey A) prior to participation in the workshop. The survey was distributed via Qualtrics Online Survey software 1 week prior to the workshop. The survey took approximately 10 minutes to complete and consisted of both qualitative and quantitative questions to assess a student's baseline confidence in time management and task prioritization skills.

Students also had 1 week to complete presession work, which took approximately 30-40 minutes. This work consisted of reading Craig E. Gordon and Steven C. Borkan's article entitled "Recapturing Time: A Practical Approach to Time Management for Physicians."[1] This article described the role of the TMMT for physician time management, how to classify tasks based on importance, and how to prioritize them based on urgency.

Copyright © 2022 Murphy et al. This is an open-access publication distributed under the terms of the Creative Commons Attribution license.

After reading this article, students were asked to complete two exercises that were specific for pediatric (Appendix B) or internal medicine (Appendix C) subinternship students. In the first part of the exercise, students were asked to develop a list of perceived clinical and personal responsibilities as well as tasks to accomplish in a typical day as a subinternship student. Students then categorized these tasks into a personalized matrix using the TMMT model. In the second part of the exercise, students reviewed specialty-specific patient cases. Each case had several tasks related to patient care that students prioritized into the matrix model.

Workshop Implementation

The workshop was held at the start of the students' subinternship rotation and hosted via videoconferencing software. Pediatric and internal medicine subinternship students participated in separate workshops. Facilitators used a PowerPoint presentation during the workshop that was specific to pediatric (Appendix D) or internal medicine (Appendix E) subinterns (speaker notes can be found for both presentations in Appendix F). The PowerPoint presentation was used throughout the entirety of the workshop to facilitate group discussion on the individual preworkshop assignments as well as additional exercises to be completed as a group during the workshop. The workshop took approximately 60 minutes and was organized into three sections.

Section 1: At the beginning of the workshop, workshop facilitators led a group discussion among student participants regarding their individualized list of clinical tasks and responsibilities created during the preworkshop exercise. Afterwards, students discussed how they categorized tasks within their personalized TMMT model. This section of the workshop, including introductions and the initial discussion, took approximately 15 minutes and corresponded to slides 1-7 of the PowerPoint presentations for both pediatric and internal medicine students.

Section 2: This section focused on reviewing the specialty-specific patient cases that students had received during the preworkshop exercise. We reviewed summaries of the patient cases as a group; then students explained how they would prioritize the patient-associated tasks and categorize them into the TMMT model. This section of the workshop took approximately 25 minutes and corresponded to slides 8-12 of the PowerPoint presentations for both pediatric and internal medicine students.

Section 3: Lastly, students participated in an exercise on triaging patient-related messages from nursing staff, which was not part of presession work. During this activity, we asked participants to place the messages into each TMMT quadrant and decide which to prioritize first based on urgency and importance. This section took approximately 15 minutes and was followed by an additional 5 minutes for a wrap-up discussion. This section of the workshop corresponded to slides 13-16 of the PowerPoint presentations for both pediatric and internal medicine students.

Evaluation

After completion of the time management workshop, we asked students to fill out a survey (Appendix A: Survey B) that took approximately 10 minutes to complete. The survey asked students to rate the quality of the workshop in improving time management and task prioritization skills and to evaluate their confidence level in these skills. We obtained additional feedback on the strengths of and areas of improvement for the workshop via free-text response. Students had 1 week to finish this survey. Students who completed the internal medicine subinternship also composed a reflective essay at the end of the rotation.

Data Collection

The surveys distributed pre- and postworkshop evaluated student confidence levels on time management and task prioritization skills. We used descriptive and nonparametric statistical analysis obtained through the Mann-Whitney *U* test to compare median confidence scores for both skills. Student essays were reviewed for comments on the time management workshop.

Results

Participants included 39 medical students from the Johns Hopkins University School of Medicine during the 2020 calendar year (May-September 2020). The response rate was 77% ($n = 30$) on the preworkshop survey and 56% ($n = 22$) on the postworkshop survey. Most workshop participants (82%) strongly agreed or agreed that the workshop improved their ability to manage time effectively and prioritize tasks on a clinical rotation. The percentage of students who reported feeling confident or very confident in time management skills increased from 40% (12) to 77% (17) after the workshop (Figure 2). The percentage of students who reported feeling confident or very confident in task prioritization skills increased from 50% (15) to 86% (19) after the workshop (Figure 3). The median time management confidence score on a 4-point scale (1 = *unconfident,* 4 = *very confident*) increased from 3.0 to 4.0 with the intervention ($p = .008$), and the median task prioritization confidence score increased from 3.5 to 4.0 with the intervention ($p = .02$; Table).

Copyright © 2022 Murphy et al. This is an open-access publication distributed under the terms of the Creative Commons Attribution license.

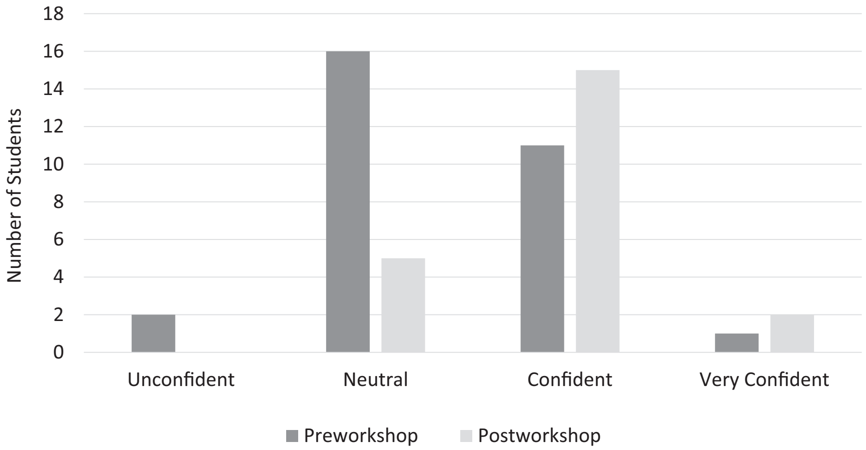

Figure 2. Students' self-assessed confidence in ability to manage time effectively on a clinical rotation. Student confidence levels were measured pre- and postworkshop.

Many students who had participated in the workshop mentioned it in their reflective essays. Of the students who mentioned the workshop, all described benefiting from the skills they had learned. One student wrote, "At first, I was unsure of how to integrate this knowledge practically into my workday. However, it soon became clear that every day presented challenges that required me to draw on the skills learned during these sessions." Another student commented, "Considering what is more important and urgent gave me mental clarity in going about my day. I have even begun using this framework for work outside of the hospital and in my personal life."

Discussion

Time management and task prioritization are essential skills for physicians. Medical schools do not often formally teach these skills as part of their curricula. There is also little evidence on the best way to effectively teach these skills. Therefore, we developed a curriculum for medical students with the goal of increasing confidence levels in these domains prior to residency.

This curriculum has shown promising initial results, as most students rated a higher confidence level in their skills upon completion of the workshop, with a statistically significant increase in the median confidence scores for both domains. However, it is unclear how this translates into clinical practice. Currently, follow-up is being conducted to survey the students after completion of their subinternship in order to evaluate the effects of the workshop in a clinical context.

The matrix model incorporated within this workshop can be an effective visual tool for individuals to assess responsibilities, evaluate time spent on completing activities, and organize

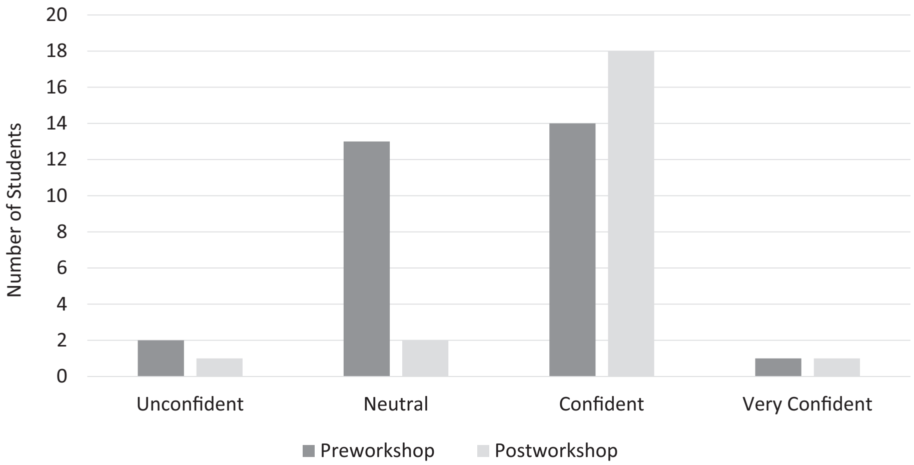

Figure 3. Students' self-assessed confidence in ability to prioritize tasks effectively on a clinical rotation. Student confidence levels were measured pre- and postworkshop.

Copyright © 2022 Murphy et al. This is an open-access publication distributed under the terms of the Creative Commons Attribution license.

Table. Participant Self-Rated Confidence Scores Preworkshop (n = 30) and Postworkshop (n = 22)

	M (*SD*) Confidence Score[a]		*Mdn* (Interquartile Range) Confidence Score[a]		
**Skill**	**Preworkshop**	**Postworkshop**	**Preworkshop**	**Postworkshop**	**Mann-Whitney *U* Test *p***
Time management	3.4 (0.7)	3.9 (0.6)	3.0 (3.0-4.0)	4.0 (3.8-4.0)	.008
Task prioritization	3.5 (0.7)	3.9 (0.6)	3.5 (3.0-4.0)	4.0 (4.0-4.0)	.02

[a]Rated on a 4-point Likert scale (1 = *unconfident*, 4 = *very confident*).

priorities accordingly. The most notable limitation for new physicians is understanding which tasks fit into each quadrant. The matrix can be difficult for beginners to use since it is very personalized and not prescriptive.

There were several additional limitations to this project, including the overall small sample size and attrition rate in survey responses. In addition, the workshop was completed at only one institution during a single calendar year. Therefore, compiling more data through implementation of the workshop during subsequent academic years as well as dissemination of the curriculum at other institutions would aid in evaluating the workshop with a wider population of medical students. It would also be beneficial to elicit data and feedback from supervisors on clinical wards to evaluate external perception of time management skills.

We learned via informal feedback received from students that, as with any initial curriculum, revisions could be made to improve the workshop. The biggest area for improvement involved increasing the fidelity of the clinical situations given to students to simulate a real-world approach to patient cases and triaging messages, which is currently underway.

Appendices

A. Student Survey Evaluations.docx

B. Preworkshop Exercise for Pediatric Students.docx

C. Preworkshop Exercise for Internal Medicine Students.docx

D. Workshop for Pediatric Students.pptx

E. Workshop for Internal Medicine Students.pptx

F. Speaker Notes for Workshop.docx

All appendices are peer reviewed as integral parts of the Original Publication.

Megan Murphy, MD: Resident, Department of Pediatrics, Johns Hopkins University School of Medicine; ORCID: https://orcid.org/0000-0002-7391-5100

Amit Pahwa, MD: Associate Professor, Department of Medicine and Pediatrics, Associate Director, Core Clerkship in Pediatrics, and Director, Advanced Clerkship in Internal Medicine, Johns Hopkins University

Barbara Dietrick, MD: Resident, Department of Pediatrics, Children's Hospital of Philadelphia

Nicole Shilkofski, MD, MEd: Vice Chair of Education, Associate Professor, and Residency Program Director, Department of Pediatrics, Johns Hopkins Children's Center

Carly Blatt, MD: Resident, Department of Pediatrics, Johns Hopkins University School of Medicine

Disclosures
None to report.

Funding/Support
None to report.

Ethical Approval
The Johns Hopkins Medicine Institutional Review Board deemed further review of this project not necessary.

References

1. Gordon CE, Borkan SC. Recapturing time: a practical approach to time management for physicians. *Postgrad Med J*. 2014; 90(1063):267-272. https://doi.org/10.1136/postgradmedj-2013-132012
2. Miles S, Kellett J, Leinster SJ. Medical graduates' preparedness to practice: a comparison of undergraduate medical school training. *BMC Med Educ*. 2017;17:33. https://doi.org/10.1186/s12909-017-0859-6
3. Peckman C. *Medscape Lifestyle Report 2017: Race and Ethnicity, Bias and Burnout*. Medscape. January 11, 2017. Accessed April 9, 2020. https://www.medscape.com/features/slideshow/lifestyle/2017/overview
4. Pitre C, Pettit K, Ladd L, Chisholm C, Welch JL. Physician time management. *MedEdPORTAL*. 2018;14:10681. https://doi.org/10.15766/mep_2374-8265.10681
5. Offiah G, Doherty E. Tricks of the trade: time management tips for newly qualified doctors. *Postgrad Med J*. 2018;94(1109): 159-161. https://doi.org/10.1136/postgradmedj-2017-135303

Copyright © 2022 Murphy et al. This is an open-access publication distributed under the terms of the Creative Commons Attribution license.

6. Covey SR. *The 7 Habits of Highly Effective People*. Simon & Schuster; 2020.
7. Brunicardi FC, Hobson FL. Time management: a review for physicians. *J Natl Med Assoc*. 1996;88(9):581-587.
8. Zackoff MW, Real FJ, Abramson EL, Li STT, Klein MD, Gusic ME. Enhancing educational scholarship through conceptual frameworks: a challenge and roadmap for medical educators. *Acad Pediatr*. 2019;19(2):135-141. https://doi.org/10.1016/j.acap.2018.08.003
9. Segers M, Van Der Haar S. The experiential learning theory: D. Kolb and D. Boud. In: Dochy F, Gijbels D, Segers M, Van den Bossche P, eds. *Theories of Learning for the Workplace: Building Blocks for Training and Professional Development Programs*. Routledge; 2011:52-65.

Received: July 19, 2021
Accepted: November 10, 2021
Published: February 22, 2022

Copyright © 2022 Murphy et al. This is an open-access publication distributed under the terms of the Creative Commons Attribution license.
